# Revisiting Recombination Signal in the Tick-Borne Encephalitis Virus: A Simulation Approach

**DOI:** 10.1371/journal.pone.0164435

**Published:** 2016-10-19

**Authors:** Yann J. K. Bertrand, Magnus Johansson, Peter Norberg

**Affiliations:** 1 Science and Historical Investigations of Evolution Laboratory of Dubá, Dubá, Czech Rep; 2 School of Medical Sciences Örebro University, Örebro, Sweden; 3 School of Natural Science, Technology & Environmental Studies, Södertörn University, Huddinge, Sweden; 4 iRiSC - Inflammatory Response and Infection Susceptibility Centre, Faculty of Medicine and Health, Örebro University, Örebro, Sweden; 5 Department of Clinical Microbiology, Sahlgrenska University, Gothenburg, Sweden; University of Padua, ITALY

## Abstract

The hypothesis of wide spread reticulate evolution in Tick-Borne Encephalitis virus (TBEV) has recently gained momentum with several publications describing past recombination events involving various TBEV clades. Despite a large body of work, no consensus has yet emerged on TBEV evolutionary dynamics. Understanding the occurrence and frequency of recombination in TBEV bears significant impact on epidemiology, evolution, and vaccination with live vaccines. In this study, we investigated the possibility of detecting recombination events in TBEV by simulating recombinations at several locations on the virus’ phylogenetic tree and for different lengths of recombining fragments. We derived estimations of rates of true and false positive for the detection of past recombination events for seven recombination detection algorithms. Our analytical framework can be applied to any investigation dealing with the difficult task of distinguishing genuine recombination signal from background noise. Our results suggest that the problem of false positives associated with low detection *P*-values in TBEV, is more insidious than generally acknowledged. We reappraised the recombination signals present in the empirical data, and showed that reliable signals could only be obtained in a few cases when highly genetically divergent strains were involved, whereas false positives were common among genetically similar strains. We thus conclude that recombination among wild-type TBEV strains may occur, which has potential implications for vaccination with live vaccines, but that these events are surprisingly rare.

## Introduction

“The hardest thing of all is to find a black cat in a dark room, especially if there is no cat.”

(Confucius).

The Tick-borne encephalitis virus (TBEV) belongs to the *Flavivirus* genus in the *Flaviviridae* family. TBEV is a positive-stranded RNA virus with a genome of about 10.5 kb that encodes all proteins in a single open reading frame (ORF), flanked by untranslated regions (UTRs). The genome is organized into structural, Capsid (C), pre-Membrane (PrM) and Envelope (E) and nonstructural genes NS1, NS2A, NS2B, NS3, NS4A, NS4B and NS5 [1]. The ORF is proteolytically cleaved into individual proteins during virus maturation.

Together with *Louping ill virus* (LIV), *Spanish sheep encephalitis virus* (SSEV), *Turkish sheep encephalitis virus* (TSEV) and *Greek goat encephalitis virus* (GGEV), TBEV forms a monophyletic group of viruses associated with ixodic hard-tick vectors [2]. The TBEV is an important human pathogen, causing chronic and acute neurological illnesses of variable severity [3]. Molecular evidence resolves the virus into three monophyletic subtypes: Western European- (W-), Far Eastern- (FE-) and Siberian- (S-) TBEV [1–4]. Phylogenies consistently associate W-TBEV together with LIV, SSEV, TSEV and GGEV. This group is sister to a clade comprised of S-TBEV and FE-TBEV. *Omsk hemorrhagic fever virus* (OHFV) is placed as sister to the whole monophyletic complex.

Within the virus’ evolutionary tree, the well resolved broad history, contrasts with the lack of detailed relationships: different branching patterns have thus been reported between TBEV subtypes and between the subtypes and other TBEV lineages [2, 5, 6–10]. Fine grain resolution has not been achieved within subtypes, except for FE-TBEV. Hypotheses for these discrepancies cover sampling artefact, differential rates of evolution between the genomic regions [6], and the fact that phylogenies have been generated with algorithms of different sophistications. A complementary or perhaps alternative process that could account for both the reported topological disparities and signal dilution is the presence of elusive genetic recombinations within TBEV.

Through recombination, several beneficial mutations that have arisen in different viral strains can accumulate inside a single genome [11], the rate of adaptation can increase [12, 13], and deleterious mutations may be expelled from otherwise functional genomes [14]. Among the most important medical aspects of viral recombination are the evolution of anti-viral resistance mutations, and the risk of using live attenuated vaccines that could recombine with other vaccine strains, or with wild-type viruses. A consequence hereof is that the vaccine strain may revert to wild type by expelling the attenuating mutations, or may create a recombinant viruses endowed with a completely new virulence. As the propensity for recombination varies widely between different viruses, a deeper understanding of the levels of recombination in TBEV is important in order to evaluate the risk of introducing live attenuated vaccine strains.

Initially described as sporadic in Flaviviruses, evidence for recombination has been accumulating in recent years. Recombination events during historical or modern evolutionary periods have been discussed in several mosquito-borne Flaviviruses: in Dengue virus [15–19], Japanese encephalitis virus [20], St Louis encephalitis virus [21, 22] and West-Nile virus (WNV) [23]. Recombination seems indeed to play a role in shaping the genomes of mosquito-borne Flaviviruses. Therefore mosaic evolution would distinguish mosquito-borne from tick-borne Flaviviruses [20], wherein evolution is clonal, with diversity generated solely by the error-prone replication with RNA-dependent polymerases. Such contrast in evolutionary dynamics was explained by possible differences in biological and ecological factors that influence viruses’ transmission [20].

However, the possibility of reticulation in TBEV was recently brought into the limelight when recombination signal was reported in all TBEV subtypes. A brief overview of the ensuing debate starts with the description of numerous recombinant strains from public databases [24]. These findings were later falsified as based on faulty alignments and unreliable genomic regions [6]. A strong recombination signal between a LIV strain and a strain from the W-TBEV subtype was reported by two different teams [6, 25] and additional weaker signals were observed in a few W-TBEV strains [25]. These reports prompted the re-sequencing of the purported recombinant LIV strain which showed that the recombination signal actually corresponded to sequencing errors or to genetic exchanges between strains during laboratory experimenting [9]. In addition, new evidence for statistically supported events in all three subtypes were unravelled. At the local level, recombination signal was revealed in a study of Slovenian W-TBEV strains, but with low statistical significance and faint phylogenetic support [26]. In sum, despite a large body of work, the presence of recombination in TBEV is still controversial as several studies have dismissed the previous reports in favor of alternative reticulation events.

All published recombination analyses have relied on the array of methods contained in the RDP package [27, 28] as part of their investigative strategies. Due to its vast popularity, this package, which combines several detection methods in a single suite, has established itself as the standard of proof for recombination inference in molecular biology. The package allows for the identification of putative recombinants, parental strains and potential breakpoints. Positive detection is reported in term of *P*-value for the null hypothesis of no recombination, and stringency is adjusted by varying the *P*-value threshold and modifying the minimum amount of agreement between methods required to validate a recombination event. During statistical detection of reticulation, a balance must be reached between detection and discriminating powers, i.e., between identifying genuine events, at the risk of hitting high levels of type I error, and distinguishing signal from noise, at the price of missing real instances of recombination at high levels of type II error.

The present contribution aims to investigate the possibility of detecting recombination within the TBEV using the RDP package. In a first phase, we simulated recombination free sequences of full length genomes, while attempting to match the empirical tree topology, divergence dates, number of strains, number of sites and rate heterogeneity among sites. Recombination free sequences provided insights into the type I error. In the second phase, we introduced a single recombination event in order to estimate the type II error.

We further applied the knowledge gained from the simulations to empirical datasets and concluded on the likelihood that the detected events captured *bona fide* recombination signals.

Finally, we compared the structure of the full genomes phylogeny with the tree derived from all publicly available E-gene sequences. We speculated on the potential to discover additional putative recombination events as more full-genome data become available.

Most phylogenetic analyses assume a single tree for the evolutionary history of a group of taxa. Recombination violates this assumption and can potentially mislead phylogenetic reconstruction in term of topology, evolutionary rates and divergence times. This has downstream consequences on studies that rely on the tree’s accuracy and can shake the conclusions about the virus phylodynamics both at the local [29, 30], and global [10, 31, 32] levels. It has further implications for our comprehension of the emergence of new strains, host and vector specificities and *in fine* virus control and vaccine development.

## Materials and Methods

### Alignments and sampling

Sequences with known collection dates were retrieved from GenBank and aligned with MAFFT v. 7.0 [33]. Full-length genomes were trimmed of the UTRs. Alignment of the ORFs was trivial due to the rarity of indels and mainly served to identify the limits of the UTRs. Sequences’ accession numbers are detailed in Table A in S1 file.

ALN1 was compiled from the ORF of 75 complete nucleotide sequences of TBEV retrieved at the time of January 2015 and divided according to strain identity into ALN1-FE, ALN1-S and ALN1-W. Each subtype alignment contained the prototype sequences (Neudoerfl for W-, Vasilchenko for S- and Sofjin-HO for FE-) from the two other subtypes to serve as outgroups. Although strains 178–79 (EF469661) and 886–84 (EF469662) were included in ALN1, they were not part of any subtype alignment because of their singular phylogenetic placements. ALN2 consists of an updated ALN1 with all new full length TBEV sequences available at the time of March 2015 and a single full length OHFV sequence. ALN3 was generated from ALN2 by removing the E-gene. ALN4 consists of all available E-gene sequences with collection dates (including the regions removed from ALN2) at the time of March 2015 of at least 1000 bp. LIV strain 369/T2 was excluded from all analyses [9]. Sequences were edited in BioEdit v. 7.0 [34].

### RDP4 settings

RDP4 v. beta 4.46 analyses were carried out with a Bonferroni-corrected *P*-value cut-off at 0.05, the disentangle recombination signals option was ‘on’ and the linear sequence setting was used. All putative recombinants were retained. Six methods were used as primary detection methods, viz. 3seq [35], RDP [36], GENECONV [37], BootScan [28], Maximum Chi Square –MaxChi– [38], Chimaera [28], and Sister Scan –SiScan– [39]. The remaining settings were kept at their default values.

### Beast analyses

The time-stamped datasets were analyzed in a Bayesian relaxed-clocked framework in BEAST 1.8.0 [40]. All alignments were partitioned by gene region. Each partition was assigned a separate substitution model as determined by MODELTEST v.3.7 [41], according to the Akaike Information Criterion. Analyses were conducted using a Bayesian skyline plot model. Alignments were partitioned by codon position with the first and second positions placed in the same category. Clock model selection was performed by running the analysis in BEAST for each of the three relaxed-clock models (strict, uncorrelated lognormal -UCLN- and uncorrelated exponential relaxed clock -UCED-) with a stepping-stone sampling (number of path steps = 100, length of chains = 10^6^) in order to compute the Bayes factors [42]. Bayes factors comparisons favored the UCLN clock model in all tested alignments.

We derived prior probabilities for the root length and substitution rates from previous studies [6]. We modeled the substitution rate priors for the UCED and UCLN clock with log-normal distributions with a mean of 1.0E-4 and a standard deviation (SD) of 1.0E-4 substitution/site/year. For the subtype alignments (ALN1-FE, ALN1-S and ALN1-W), the root age priors were modeled using normal distributions with a mean of 2000 yr and a SD of 750 yr. All other alignments included some OHFV strains and had therefore an older root age that was modeled with a normal prior (mean = 5000, SD = 1000) in order to approximate the highest posterior density regions at 95% (95% HPD) of [2449–7137] obtained from ref 32.

For each analysis, four independent MCMC chains were run for 50 x 10^6^ generations and their log outputs combined with 10% burn-in samples discarded. Maximum clade credibility trees (MCC) trees were summarized with TreeAnnotator [40]. Tracer v.1.5 [43] was used to determine degree of mixing, shape of the probability density distribution, median and HPD intervals for the relevant parameters. Adequacy of sampling was assessed via effective sampling sizes (ESS always exceeded 200 for the investigated statistics) and mixing.

We used a two steps BEAST analysis in order to achieve accurate dating for the E-gene alignment (ALN4) [6]. Briefly, estimates of posterior substitution rates appear upwardly biased for the E-region when compared with other portions of the genome and, as a consequence, divergence dates retrieved from E-sequences alone tend to be younger. In order to compensate for this bias, we obtained posterior substitution rate estimates and posterior distributions for two divergence events (the root of the (FE-,S-) clade and the root of the (W-, LIV, TSEV, SSEV) clade) from full-length genomes without the E-region (ALN3). These posterior distributions were used as priors in the analysis of the E-sequences data (ALN4). The modes and parameters of the posterior distributions were estimated using the distribution fitting software EasyFit 5.3 (MathWave Technology) and modeled with gamma distributions.

### Simulation preprocessing

**Fig 1:**
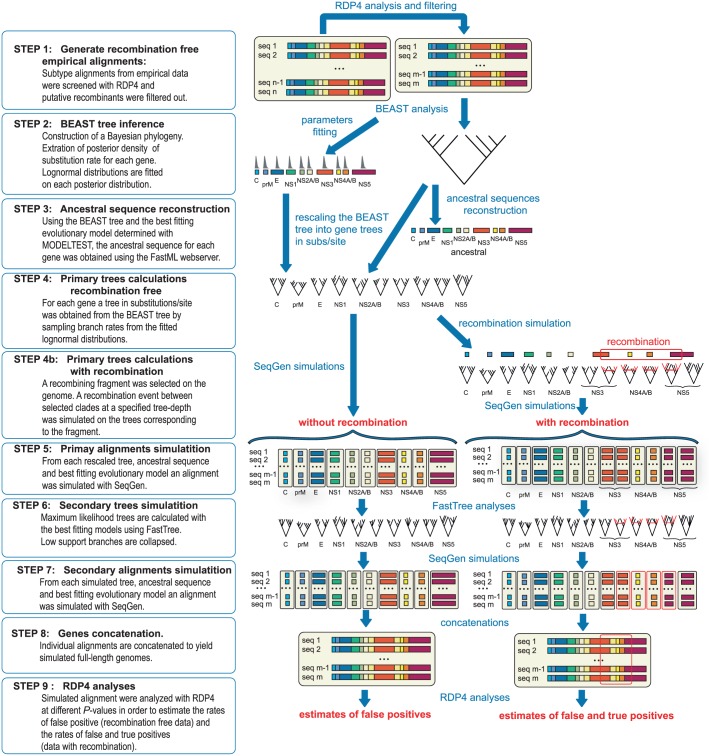
Synoptic diagram presenting the methods and analytical framework deployed during in the simulation. The analytical protocol designed to estimate the rates of true and false positive for the detection of recombination in simulated data consists in several steps: STEPS 1–3 derive the parameters for the simulation from empirical sequence alignments. STEPS 4–8 simulate alignments that are similar to the empirical data in term of tree topology, divergence dates, number of strains, number of sites and rate heterogeneity among sites. Several stochastic processes are added in order to model lineage specific substitution rate variation (STEP 4) and model the effect of purifying selection (STEP 6). The simulated datasets with and without recombinations are finally analyzed with RDP4 (STEP 9).

See Fig 1 for an overview of the simulation protocol. Subtype alignments (ALN1-FE, ALN1-S and ALN1-W) were screened for recombination and putative recombinant strains were filtered out (see results) if the signal was statistically significant (*P*-value = 0.05) and all strains in a recombination triplet (recombinant and parental strains) were identified (STEP 1). The alignments were analyzed in BEAST and the MCC trees were summarized. For each gene, we extracted the posterior substitution rate values, fitted a lognormal distribution with EasyFit and recorded the values of μ and σ (STEP 2). For each subtype, we reconstructed the ancestral nucleotide sequence from the MCC tree, the alignment and a GTR substitution model using the FastML webserver [44] (STEP 3).

### Simulating recombination free alignments

For each subtype, we first simulated recombination free alignments. The gene was considered to be the unit of selection, thus each gene region was endowed with its own genealogy obtained by modifying the MCC tree derived from the entire genome as explained below. The general simulation strategy was:

1. Model lineage-specific substitution rate variation.
2. Model variation in substitution rates across the genome, which simulates the effect of variable strengths of purifying selection. Simulate the effect of stochastic mutational variance.
3. Evolve nucleotides on simulated genealogies that account for the processes i and ii.

#### Model variations in substitution rates across lineages and across the genome

For each gene, variations (i) and (ii) were implemented simultaneously: the MCC tree in generation unit was rescaled into substitutions/site by multiplying each branch length by a substitution rate. The rate value was sampled from a lognormal distribution with shapes parameters (μ, σ) inferred from the empirical distribution, thus producing a rescaled tree, called primary tree (STEP 4).

#### Simulate the effect of stochastic mutational variance

Stochastic mutational variance refers to the random conflict in the data derived from repeated mutations in the absence of recombination. For each gene, a primary sequence alignment was evolved from the inferred ancestral sequence on the primary tree using the empirical model of nucleotide substitution. Primary alignments that reflected the empirical data in term of length and number of sequences were generated in SeqGen v1.3.3 [45]. Trees were constructed from the primary alignments under the GTR model using FastTree 2.0 [46]. Splits reliability was estimated using the Shimodaira-Hasegawa (SH) [47] test on the alternate tree topology around each split and used as a measure of branch support (STEP 5). A secondary tree was obtained by collapsing branches with support below 0.7 and randomly resolving the polytomies (STEP 6). Secondary alignments were evolved on the secondary trees as previously (STEP 7). Because secondary trees were different from primary trees, we broke a possible vicious circle that could have been induced by using RDP4 for simulating recombination free data and subsequently analyzing these data with the same tool.

### Simulating alignments with recombination

**Fig 2:**
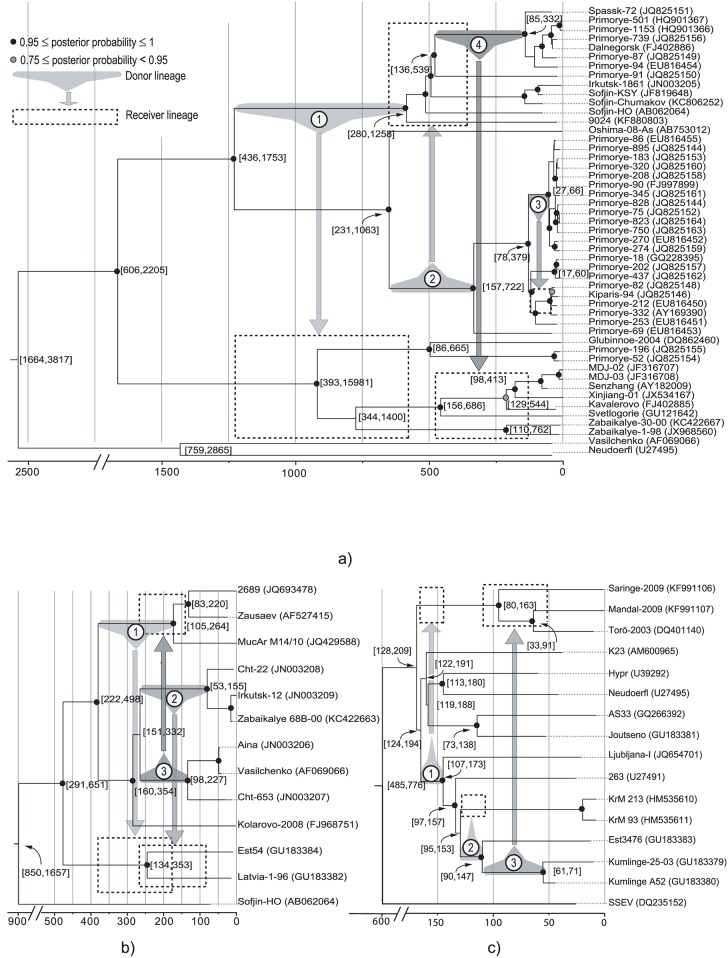
Selected lineages for simulating recombination events in the three TBEV subtypes: FE- (a), S-(b) and W-(c). For each strain the corresponding GenBank accession number appears in parentheses. Trees have been derived from BEAST analyses using the time stamped ALN1-FE, ALN1-S and ALN1-W alignments. For some selected nodes, the 95% HPD for their divergence times is shown between square brackets. All dates correspond to years before present. Posterior probabilities of branches are indicated by coloring the nodes in different shades of grey. Uncolored nodes bear branch support values below 0.75.

We simulated a single recombination event per alignment at different tree depths and investigated various lengths for the recombination fragments. Three recombination events were studied for the S- and W- subtypes and four for the FE-subtype; their locations in the trees are depicted in Fig 2. For each event, the branch yielding the minor parental strains was called the donor lineage and the tree subpart derived from this branch, the donor clade. The clade including the major parental strains was the receiver clade. In order to maximize the power of the recombination detection, lineages were selected so that recombination could change the topology of the induced tree.

For each fragment size (*l*)-recombination event pair, we randomly selected the position of the fragment along the genome, with the 5´ breaking point located on the region spanning 0 to *length of genome—l*. A recombination event was then simulated on the primary tree corresponding to this genome region. We randomly chose a point on the donor lineage and identified a point in a branch of the receiver clade that was placed at the same time from the most recent common ancestor (MRCA) of the donor and receiver clades. The branch was pruned from that point and re-graphed at the donor point, thus yielding a recombinant primary tree. Therefore, only the genome portion carrying a recombinant fragment had a different evolutionary trajectory from the remaining genome which still evolved according to the primary tree. When the recombinant fragment spanned several genes, the timing of the recombination and the identity of the donor and receiver clades were common to several recombinant primary trees, but each of them was derived from a primary tree corresponding to a different gene region. In addition, when a recombinant event split a gene into several fragments, the recombinant and non-recombinant regions of the gene were associated with different primary trees (STEP 4b). Primary alignments were evolved on the recombinant and non-recombinant primary trees and analyzed with FastTree to yield secondary trees with and without recombination. Finally, secondary alignments were simulated from the secondary trees. For each event, we recorded the location of the breakpoints in the genome and the duration from the recombination time to the time of the MRCA (tMRCA). Topological perturbations were assessed with the Robinson-Foulds (RF) [48] distance between non-recombinant and recombinant primary trees. All secondary alignments were concatenated into one simulated genome (STEP 8).

For each combination of variables, we simulated one hundred alignments. We investigated four recombination lengths (200, 1000, 2000 and 3000 bp) for ten events across all three subtypes. Before analysis in RDP4, outgroup sequences were removed from the simulated alignments. For a simulated recombinant fragment of length *l* inserted between breakpoints *b1* and *b2* to be deemed correctly detected, it had to be found in the genomic region spanning *b1 –l* to *b2 + l* and all sequences from the triplet (putative recombinant, minor parent, major parent) had to be discovered in the receiver and donor lineage with the putative minor and major parent placed in different lineages (STEP 9).

### Dating the empirical recombination events

Five strains from the FE-subtype displayed a strong signal (see results section) that could proceed from genuine recombination events given the criteria established by the simulation. In order to date these events, a new alignment was generated from the ALN2 by removing all non FE-strains except for 178–79 and 886–84 that served as outgroups and deleting the five putative recombinant strains. Then, for each event, the putative recombinant strain was added in turn to the alignment, which was partitioned into a recombining and a non-recombining fragment. We subjected the resulting alignments to BEAST analyses parameterized as above. The root prior was modeled with a normal distribution (mean = 3000, SD = 1000). For each pair of alignments, the MCMC chain was run for 50M generations, sampled every 5000. In the neighbor joining tree obtained in RDP4, the two recombining fragments located in the Primorye196 (JQ825155) strain branched at similar places and were thus considered as derived from the same recombination event.

### Assessing phylogenetic signal supporting the empirical recombination events

The five strains with strong recombination signals were further subjected to the SH-test at *P* = 0.05. We carried out a reciprocal SH-test, by first enforcing the tree topology from the recombining fragment on the non-recombining data and then constraining the non-recombining topology on the recombining data. We used RAxML v.7.8.8 [49] to compute the maximum likelihood (ML) trees under the GTR+Γ model and perform the SH-tests.

The simulation was implemented in a Python 2.7 pipeline built from the Dendropy [50], ETE [51] and Biopython [52] libraries. Analyses were performed on the bioinformatics computer cluster Albiorix at the Department of Biological and Environmental Sciences, University of Gothenburg.

## Results

### Empirical data

RDP4 reported numerous recombination events in the FE- and S- subtypes (16 and 22 respectively), whereas only three events were inferred in W- (Table 1). The highest levels of statistical support were obtained in the FE-subtype with four events detected by all methods (events 1, 2, 3 and 5).

**Table 1:** Results of the recombination analyses using RDP4 applied to empirical data (W-, S- and FE-ALN2 alignments) at detection *P-*value of 0.05.

							Detection methods						
Subtype	Event	Start	End	Recombinant(s)	Minor parent(s)*	Major parent(s)	3Seq	Bootscan	Chimaera	GenConv	Maxchi	RDP	SiSscan
**FE**	1	2902	6848	Shkotovo-94	Primo212, Primo183, Primo18, Primo202, Primo208, Primo253, Primo270, Primo274, Primo320, Primo332, Primo345, Primo437, Primo69, Primo7503, Primo75, Primo823, Primo828, Primo82, Primo86, Primo895, Primo909, Kiparis-94,	Primo94, 1230, Dalnegorsk, Irkutsk-1861, Primi89, Primo1153, Primo2239, Primo501, Primo633, Primo7396, Primo87, Primo91, SofjinKSY, Sofjin-Chumakov, Sofjin-HO, Spassk-72	1.5E-29	7.9E-45	3.5E-19	1.3E-37	1.0E-21	8.9E-39	5.8E-30
	2	3796	4839	Primo196	Primo1153, 1230, Dalnegorsk, Irkutsk-1861, Primi89, Primo2239, Primo501, Primo633, Primo739, Primo87, Primo91, Primo94, SofjinKSY, Sofjin-Chumakov, Sofjin-HO, Spassk-72	Primo52, Glubinnoe-2004	1.4E-16	2.1E-27	3.1E-11	5.7E-25	6.4E-11	2.7E-27	1.3E-12
	3	6867	7806	Primo196	Primo1153, 1230, Dalnegorsk, Irkutsk-1861, Primi89, Primo2239, Primo501, Primo633, Primo739, Primo87, Primo91, Primo94, SofjinKSY, Sofjin-Chumakov, Sofjin-HO, Spassk-72	Primo52, Glubinnoe-2004	1.2E-18	5.5E-31	1.4E-09	2.0E-28	1.0E-09	6.5E-31	5.0E-11
	4	5541	5946	Primo52	Sofjin-HO, 1230, Primi89	Primo196, Glubinnoe-2004		7.3E-08		2.7E-06			
	5	435	990	Glubinnoe-2004	205	Primo196, Primo52	1.4E-02	2.4E-06	1.8E-04	6.7E-05	3.9E-03	2.8E-06	1.4E-04
	6	6488	6671	205, 9024	Primo69, Kiparis-94, Primo183, Primo18, Primo202, Primo208, Primo212, Primo253, Primo270, Primo274, Primo320, Primo332, Primo345, Primo437, Primo750, Primo75 Primo823, Primo828, Primo82, Primo86, Primo895, Primo909, Shkotovo-94	Primo2239				1.16E-03			3.3E-05
	7	8343	9123	MDJ-03, MDJ-02	Xinjiang-01	Senzhang		1.4E-04		9.1E-03	1.92E-03	1.5E-04	
	8	7952	8342	MDJ-01, Senzhang	MDJ-02, MDJ-03	Xinjiang-01	1.5E-03	3.6E-08		3.6E-06			
	9	8343	9294	MDJ-01	Senzhang, WH2012	Xinjiang-01, MDJ-03		2.3E-05					
	10	6690	9219	Primo501, Primo1153	Primo2239	Primo87		1.1E-03			1.1E-04	4.6E-02	6.3E-04
	11	2190	2613	Glubinnoe-2004	Sofjin-HO, Dalnegorsk, Primo196, Primo1153, Primo2239, Primo501, Primo633, Primo739, Primo87, Primo91, Primo94, Sofjin-Chumakov	Primo52		1.9E-03	1.5E-03		2.7E-02	3.3E-03	
	12	6462	6550	Primo633	Zabaikalye	Sofjin-Chumakov, Irkutsk-1861, Primi89, Primo91, SofjinKSY		3.0E-03				3.4E-03	
	13	3381	3413	MDJ-02	Primo52	MDJ-03				8.7E-03			
	14	9155	9541	Glubinnoe-2004	Primo2239	MDJ-02, MDJ-03							3.2E-02
	15	4536	4913	Primo253	Svetlogorie	Primo94		3.2E-02					
	16	9338	10105	Xinjiang-01	Svetlogorie	WH2012	3.4E-02				1.8E-03	2.4E-05	
**S**	1	5200	5244	2689	Est54, Latvia-1-96	MucAr-M14-10, Aina, Cht-653, Kolarovo-2008, Vasilchenko, Lesopark, IR99-22f7, Irkutsk-BR-683-11				2.8E-05		4.7E-06	
	2	6375	6459	Zabai	Vasilchenko, Aina	Irkutsk-12		2.0E-05		1.0E-04			
	3	3784	4075	Kolarovo-2008	MucAr-M14-10, 2689, Zausaev	Cht-22, Cht-653, Irkutsk-12		2.2E-02	7.5E-03		1.6E-02		1.2E-04
	4	8566	8718	Kolarovo-2008	Zausaev	Cht-653						8.6E-03	1.9E-04
	5	108	1284	Kolarovo-2008	Zausaev5	Cht-22, Vasilchenko		1.0E-02			3.0E-03		
	6	7645	7867	Kolarovo-2008	2689	Cht-22		3.4E-02					
	7	7280	8215	Zausaev	2689	MucAr-M14—10		2.6E-09	1.6E-05	4.1E-07	9.0E-05	8.5–04	
	8	1998	2301	Zausaev, MucAr-M14-10	Latvia-1-96,	Irkutsk-12, Aina, Cht-653, Sakhalin-6-11, Vasilchenko							1.3E-02
	9	8562	9550	Kolarovo-2008	Irkutsk-12, Cht-22, Sakhalin-6-11, Zabaikalye-1-09	Vasilchenko, Aina		5.7E-04					4.4E-08
	10	7187	8199	Kolarovo-2008	Cht-653, Aina, Irkutsk-BR-683-11	Tomsk-PT122			2.0E-05		2.1E-04		
	11	587	1174	Kolarovo-2008	IR99-22f7, Lesopark 11, Zausaev	Tomsk-PT122			4.7E-02		2.5E-05	2.4E-03	
	12	4518	5226	Aina, Cht-653, Vasilchenko	Buzuuchuk	Tomsk-PT122, Kolarovo-2008, MGL-Selenge-13-12		6.3E-07	2.9E-05	3.4E-06	7.2E-06	2.4E-07	1.9E-08
	13	3860	4258	Vasilchenko, Aina, 653, Tomsk-PT122	Buzuuchuk	Irkutsk-12, Sakhalin-6-11		5.4E-05		1.7E-03	1.0E-02	1.8E-03	1.2E-05
	14	5805	6240	Vasilchenko, Aina, Cht-653	Buzuuchuk	Tomsk, Kolarovo-2008		9.4E-06	2.4E-03	3.1E-04	2.9E-03	1.2E-05	4.1E-05
	15	3525	3859	Irkutsk-BR-683-11, Cht-22, Irkutsk-12, Sakhalin-6-11, Zabaikalye-1-09	2689	Aina, Cht-653, Vasilchenko		6.8E-04				7.92E-04	
	16	1701	2250,	Buzuuchuk	Aina, Cht-22, Cht-653, Irkutsk-12, MGL-Selenge-13-14, Sakhalin-6-11, Tomsk-PT122, Vasilchenko, Zabaikalye-1-09	IR99-22f7, 2689, Lesopark, Zausaev				4.7E-02			5.7E-07
	17	9454	9894	Buzuuchuk, Tomsk-PT122, Vasilchenko	Aina	IR99-22f7, MucAr-M14-10, Zausaev							1.5E-04
	18	7050	7635	Buzuuchuk	Aina	MucAr-M14-10, Lesopark, Zausaev				7.0E-03	4.5E-02		4.9E-04
	19	8030	8624	Buzuuchuk	Aina, Cht-653, Vasilchenko	2689, Lesopark, MucAr-M14-10		6.3E-03				3.0E-03	3.1E-06
	20	3926	4155	Latvia-1-96	Buzuuchuk	Lesopark, IR99-22f7, MucAr-M14-10					8.7E-03		1.0E-04
	21	5091	5564	Kolarovo-2008	MGL-Selenge-13-12, Cht-22, Irkutsk-12, Irkutsk-BR-683-11, MGL-Selenge-13-14, Sakhalin-6-11, Zabaikalye-1-09	Tomsk-PT122		5.0E-04	2.8E-02			8.9E-03	1.4E-03
**W**	1	946	1702	Joutseno	Est3476	AS33		1.3E-05	3.9E-02	6.0E-04	6.7E-04	4.1E-03	3.5E-07
	2	6997	8593	Joutseno, AS33	Est3476	Stockholm, Norwegian, Toro-2003		1.3E-07	4.57E-02	1.7E-07	1.5E-06	3.2E-02	2.0E-11
	3	4719	5836	Stockholm, Norwegian, Toro-2003	Kumlinge-A52	Ljubljana-I					8.1E-03		

*The prefixes “Primo” and “Primi” refer to the strains called “Primorye” and “Primirye” respectively.

The start of ORFs is used as the origin for reporting the location of recombination breakpoints along the alignment.

### Simulated alignments

#### False positive rates in absence of recombination

For the simulations without recombination, we recorded the absolute number of false positives for 100 replicates and the mean per replicate (Fig A in S1 file). False positive rates greatly exceeded the 5% expectation, with large variations depending on the method and the targeted subtype. The highest levels of false positive were obtained in the W- (mean above 8 events per replicate), followed by the S- (5.5) and were the lowest in the FE-subtype (3.5). Lowering the detection *P*-value and requiring several methods to concur decreased the false positive rates. In the FE-subtype, it was below 5% when we enforced the agreement of at least 2 methods at a *P*-value of 1.0E-6. For the S-subtype, levels of false positive approached 5%, at a *P*-value of 1.0E-9 with the agreement of at least two methods. For the W-subtype, the 5% threshold was reached with three agreeing methods at 1.0E-6 and with at least two agreeing methods at 1.0E-9. Across all analytical settings, 3Seq and GENECONV displayed consistently the lowest false positive rates. Among the other methods, BootScan demonstrated the lowest rate, whereas MaxChi, Chimaera, and SiScan had the highest and reported very similar false positive rates.

#### True and false positive rates in presence of recombination

Results of the simulations with one recombination event are reported in Figs B-G in S1 file, which display the absolute number of true and false positive detections for 100 replicates. Tables B-J in S1 file give the mean false positive rates per replicate. For each simulated event, the range of durations between the recombination tMRCA of the donor and receiver clades are provided in Table 2. Based on these intervals, the simulated events were ascribed to four partially overlapping categories: deep (FE- event 4), intermediate-deep (FE- events 1 and 2), intermediate-shallow (S- events 1, 2 and 3) and shallow (FE- event 3 and W- events 1, 2 and 3). These durations correlated with sequence dissimilarity between the minor and major parents.

**Table 2:** Range of durations between the recombination tMRCA of the donor and receiver clades for the simulated events.

Subtype	FE				S			W		
event	1	2	3	4	1	2	3	1	2	3
Sampled durations	[435,1077]	[578,894]	[0,74]	[1187,1524]	[304,421]	[211,302]	[115,207]	[20,26]	[0,17]	[98,151]

Table displaying for each simulated recombination event, the range of sampled durations in years between the time of the recombination event and the tMRCA of the donor and receiver clades. tMCRA is set to the median age of the split between the two clades based on posterior density regions at 95% for the node ages in the empirical BEAST trees from subtype alignments (ALN1-FE, ALN1-S and ALN1-W).

Adding a single recombination event modified the propensity for false positives, which reached higher levels than in absence of recombination. Neither the length of the recombining fragment nor the identity of the recombination event had an appreciable effect on the false positive rate, which depended mainly on the subtype identity.

Methods with low false positive rates (3Seq and GENECONV) displayed lower true positive rates and symmetrically methods with high detection power had high false positive rates (BootScan, MaxChi, Chimaera, and SiScan).

For deep and intermediate-deep events, a fragment size of 200 bp was barely detected, usually around 20%, but with four fold false positive rates, when no agreement between methods was enforced at *P*-value of 0.05. The true positive rate plummeted with the increase of stringency. For intermediate-shallow and shallow events, no simulation could detect the recombining fragment in more than a few replicates.

Detection was optimal for the deep event (FE- event 4) involving all fragments longer than 200 bp (Fig C in S1 file). All stringency levels yielded a detection rate above 95%, which means that stringency could be varied to reach admissible false positive rates. Thus, for a *P*-value of 1.0E-6 while requiring two methods to agree (and for all higher stringency levels), the false positive rate was below 5%.

Detection rate was lower for the intermediate-deep events (FE- events 1 and 2) in Fig B in S1 file. Decreasing the detection *P*-value to 1.0E-6 with the concordance of at least two methods allowed to reach false positive rates below 5% while keeping acceptable positive rates (usually above 50%) for fragments longer than 200 bp.

For intermediate-shallow events (events in the S-subtype in Fig D in S1 file) the recombinations were well recovered only for the 3000 bp fragment at the lowest detection *P*-value, but at the cost of unacceptable false positives. False positive rates stayed consistently above 5% and could only be lowered to around 20% in the most stringent conditions for S- events 1 and 3, in which case the true positive rate did not reach above 10%. For S- event 2, all considered detection stringency produced false positive rates that exceeded true positive rates.

Shallow events (FE-event 3 in Fig C in S1 file and all W- events in Figs F-G in S1 file) did barely register irrespectively of the detection strategy.

#### Relationship between strength of detection and phylogenetic location of the recombination events

The results showed a clear trend linking detection power with the duration (*d*) between the time of the recombination event and the tMRCA. However, the strength of the relation varied greatly with the length of the recombining fragment. We applied a log10 transformation to the inverse (f(x) = 1/x) of the *P*-value data associated with positive detection. We investigated the strength of the correlation between transformed *P*-values and the duration (*d*). Using Pearson product-moment correlation coefficient (ρ), the strongest correlations were obtained when *d* was combined with the RF distance, i.e., with a composite variable (θ), product of the duration and the topological perturbation. Weak correlations were observed (ρ <0.5) for fragments of 200 and 1000 bp (data not shown). The strongest correlations were obtained for 2000 and 3000 bp fragments when three methods had to agree. Transformed *P*-values plotted against their corresponding θ are reported in Fig H in S1 file for the two largest fragments. The scatterplots clearly demonstrated variable responses to the same event from different detection methods. 3Seq, Bootscan, GENCONV and RDP had stronger responses than MaxChi, Chimaera and SiScan. Chimaera displayed the largest dispersion and lowest correlation. For θ larger than 6000 all methods reported a majority of responses stronger than 1.0E-6.

#### Phylogeny of E-sequences

**Fig 3:**
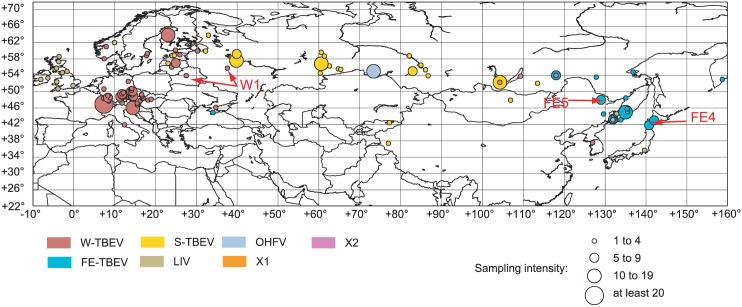
Collection localities for the E-sequences used to build the tree in Fig I in S1 file. Strains origins for three clades of interest (W1, FE4 and FE5) are indicated with arrows. Because strain origins are reported with various levels of precision (from local to national level), this map should only be used as an indication of the patchy record of TBEV genetic diversity. This map also shows that the territory comprised between the Irkutsk and Zabaikalsky regions represent a hot spot of genetic diversity with the co-circulation of X1-, X2-, FE- and S-strains. Some well known foci such as the presence of the S-TBEV in Finland and the isolation of all three sub-types in Estonia and Latvia are not included on the map as the associated sequences are too short to yield reliable phylogenetic signal. Sampling intensity is given in number of sequences collected in the same locality. The map was generated using an equidistant cylindrical projection with the Basemap toolkit available from the python package Matplotlib (http://matplotlib.org/basemap/users/cyl.html?highlight=cylindrical).

Topology and divergence dates in the E-sequences MCC tree (Fig I in S1 file) were mainly in line with previously published works [6, 9, 31], except for the divergence of the FE-subtype that was pushed back in time due to the influence of the large amount of S-strains on the coalescence inference. The lack of a fully coherent geographical structure at the fine scale demonstrated a potential for large distance dispersal [53], as evidenced by the sampling localities plotted on Fig 3. Indeed, in many instances, strains that clustered together in the E-tree have been sampled several hundreds of kilometers apart. The purpose of the E-tree was to reveal new divergent lineages that cannot be currently tested for recombination due to the lack of full genome sequence. We could then compare this phylogenetic diversity with the one used in the simulation to speculate about our ability to detect a recombination event in these new lineages.

The E-tree indicated that the known phylogenetic diversity is overall remarkably well sampled by the currently available full genomes. The tree revealed some clades displaying a low full-genomes/E-sequences ratio, such as the large S2 and S3 clades and most of the W2 clade. Our simulation demonstrated that under some circumstances, recombination detection between S- subclades was possible, which pleads for additional genome sequencing efforts for S2 and S3 strains. Such efforts are especially important for S3 that corresponds to the Baltic clade [54], whose strains co-circulate with FE- and S- strains in the Baltic region, hence increasing the possibility of inter-subtype recombination.

Although there was a considerable disequilibrium between known strains and sequenced genomes in W2, adding more genome would not lead to additional detection of recombination between W2 strains. However, two strains (Pan AF091015 and 70 KF573601) formed a distinct clade W1, which diverged from W2 around 900 years ago (95% HPD of [540, 1059]) and should possess enough sequence dissimilarity for identifying a potential recombination event between a W1 and a W2 strain. Finally, two divergent lineages in the FE-subtype, FE4 (Kam586/97 AB237185 and Kam588/97 AB237186) and FE5 (DXAL-18 EU089979) should harbor sufficient dissimilarity to allow detection of a putative recombination with other FE- strains. Additional sequencing efforts should thus target these few TBEV clades where reliable recombination signal could be identified.

#### Reappraisal of detected recombinations in empirical data

According to our simulations, recombination signal was overwhelmed by noise for W-strains, therefore none of the reported recombination within this subtype are reliable.

Similarly, short recombinations (<1000 bp) within the S-subtype were not trustworthy. None of the putative event fulfilled the requirements for observing *bona fide* recombination in the S-clade (i.e., *P*-values below 1.0E-9 and the agreement of at least three methods).

Within the FE-subtype, six putative events met the minimum criteria for genuine recombination (see Table 3), i.e. a minimum length around 1000 bp and the agreement of at least two methods at 1.0E-6. Each event was dated with BEAST (see Fig J in S1 file). Because events 2 and 3 involved the same parental and recombinant strains, they were likely to result from the same event through multiple crossing-over. Although RDP4 identified an identical recombinant signal in two strains for event 8, differences in tree topology demonstrated that they could have originated from separated events. All recombination events were corroborated by phylogenetic evidence, with high supports for the nodes producing discordant topologies. All events belonged to the deep category except for event 8 (intermediate-deep). Events from this category had a high rate of true positive and a low rate of false positive for fragments of at least 1000 bp. We assessed phylogenetic discrepancies between the recombining and non-recombining fragments using a reciprocal SH-test. A significant difference between the two trees was detected in both tests in all cases, except for event 5. In event 5, the two trees were significantly different when the non-recombining tree was constrained by the recombining one, but not in the reciprocal test. This result points to a moderate phylogenetic signal in the recombining fragment. This test offers thus further evidence that event 1, 2 and 3 correspond to reliable recombination signals. Despite some phylogenetic evidence, highly significant detection in RDP4 and high sequence divergence, we lack simulation data for fragments of length 390–555 bp to reach a definitive conclusion on the trustworthiness of events 4, 5 and 8.

**Table 3:** Reappraisal of the RDP4 identified putative recombination events.

	Event	Length (bp)	Region	No recombination mean node age (95% HPD)	Recombinationmean node age (95% HPD)	tMRCA mean node age (95% HPD)	Duration: tMRCA to recombination event	Simulation category
Subtype FE	1	3946	NS1-NS4B	646 [450,973]	41 [25,65]	1071 [749,1579]	1030	deep
	2	1043	NS2A-NS3	920 [620,1542]	100 [39,147]	1294 [818,1922]	1194	deep
	3	939	NS4B-NS5	-	-	-	-	-
	4	405	NS3	1292 [721,1696]	106 [75,271]	1574 [1016,2254]	1468	deep
	5	555	PrM-E	1358 [793,2060]	274 [63,464]	1597 [1063,2495]	1323	deep
	8	390	NS5	MDJ-01: 58 [18,88], Senzhang: 190 [79,182]	MDJ-01: 6 [2,49], Senzhang: 70 [59,235]	488	MDJ-01: 482, Senzhang: 412	intermediate-deep

The events in subtype FE correspond to those presented in Table 1. Event 2 and 3 are considered to be derived from the same event. The length of the recombining fragment and the genome region spanned by the fragment are indicated. The “no recombination mean node age” refers to the divergence date for the recombining strain in the recombination free alignment, whereas the “recombination mean node age” reports the divergence time of the strain in the alignment region covering the recombination. The time of the most common recent ancestor of the parental strains that produced the recombinant (highlighted by a red circle in (Fig J in S1 file) is given by the tMCRA. The tMCRA was calculated on the largest alignment, that is, on the recombination free sequences. The duration was calculated as the time from the tMRCA to the time of the recombination event. All dates and duration are reported in years. Events are assigned to simulation categories based on their duration values.

## Discussion

We have established that some events in the FE-subtype are supported by reliable recombination signals. In order to rule out laboratory induced events, the strains associated with the recombinations need to be re-sequenced. However, in absence of the original viral extract, re-sequencing would not necessarily reveal an artificial recombinant obtained from template switching as the result of *in vivo* or *in vitro* experiments carried in a laboratory handling different subtypes or in presence of strains from different subtypes in the original extract [9]. In such cases, a genuine recombination signal would only be ascertain by discovering new strains harboring the same signal. Furthermore, we have identified several sufficiently divergent lineages in the FE- and W- clades, known only through their E-sequences, where putative recombinations, if present, could be reliably detected in full-genome alignments.

Additionally, we suggest that many of the previously reported events could correspond to false positives and that the rate of recombination in TBEV has been overestimated. Contrary to previous reports, when genuine signal is disentangled from noise, recombination emerges as surprisingly rare.

### Assumptions of the simulation

Previous works based on simulated and empirical data have evaluated detection strategies in terms of absolute and relative performances by thorough explorations of the parameter space. Such space consists of level of recombination, genetic diversity, and rate variation among sites [55], amount of subsequent substitution after a recombination event [56], or tree topology and depth retrieved from published phylogenies [55]. Contrasting with these large-scale studies, we approached the problem by intensively studying a single case with its idiosyncratic phylogenetic setting and sequence variation, which meant deriving all simulation parameters from empirical data.

Our protocol rests on assumptions that need to be carefully examined. Because we simulated alignments from a tree model and conclude that the empirical data contains few recognizable ancient recombination events, i.e., that the evolutionary history is mostly tree-like, it could appear that we were begging the question, which means assuming as premises the conclusion of the argument. Although we posit a tree-like evolution based on available evidence we do not conclude that recombination is rare overall. On the contrary, it might be very common between closely related strains within the same geographic region. We demonstrate instead that there are few reliable recombination events among the detected signal in the empirical alignments.

We further assumed the random distribution of recombination breakpoints along the genome, as observed in the empirical data. We limited our model to a single recombination per replicate in order to avoid complex interactions between multiple signals. In most cases, the tree inferred from the recombining region was different from the tree from the non-recombining in order to maximize the detection power. Hence, the reported detection rates might not fully reflect the rates obtained in absence of topological incongruence. We introduced three stochastic factors that might confound recombination detection: variation in substitution rate across lineages, variation in substitution rate across the genome and stochastic mutational variance. Stochastic mutational variance aims to capture the substitution model error due to the model’s failure to account for the complexity of the data. Such variance was obtained by generating trees from each gene region and collapsing branches with support lower than a given score. This score was set to a level that produced alignment with similar rescaled consistency index [57] to the empirical data (RCI ~ 0.6). However, because the empirical data are likely to contain undetected recombination signal, the simulated data might harbor more stochastic variation than what can be obtained in real sequences in absence of recombination. Therefore, our simulation might be overestimating the rate of false positive in empirical data, which makes our results a conservative estimate of the reliability of the detected recombination.

We modeled four recombination fragment lengths present in the empirical data. The detection power increased with length. Fragment above 200 bp produced high detection rates under some conditions, whereas at the length of 200 bp detection was low at any detection stringency. These results entail that events involving recombining fragment measuring between 200 and 1000 bp lay in a gray zone in term of detection reliability. In addition, if a recombining fragment larger than 3000 bp were to be detected in the W-subtype, additional simulations would be necessary to conclude on its trustworthiness.

### Low substitution rates in TBEV

Compared to the extensive simulations in ref 55, we have obsersved a much lower detection power, which could be explained by differences in sequence similarity. In ref 55, a minimum sequence divergence of 5% was necessary to obtain substantial power. Whereas, sequence divergence (as measured with uncorrected p-distance) was much lower in the W-alignment (mean = 2.5E-2, SD = 5.0E-3), slightly below 5% in the S-alignment (mean = 4.2E-2, SD = 2.0E-2) and barely reached above 5% only in the FE-alignment (mean = 6.1E-2, SD = 3.7E-2). Therefore, the low recombination rate in TBEV might reflect our difficulties to detect recombination at low substitution rates.

### Validity of the detected recombinations

When searching for small recombining fragment in noisy data, the default 0.05 detection *P*-value in RDP4 does not correspond to a probability of 5% that the putative event represents a false discovery. Indeed, at such threshold, true positives are swamped with false negatives. We showed that although reliability increases when several lines of evidence were combined, the interpretation of the reported detections requires taking the phylogenetic context, the amount of sequence overprinting since the recombination time, the specific method and fragment length into consideration when reaching a conclusion on the reality of the event.

### TBEV evolutionary dynamics

We demonstrated the difficulty to identify recombination events between closely related strains, events which due to the strains close spatial proximity are the most likely to occur. To fully appreciate the true rate of recombination, fine grained population studies at the local level will be required. Additionally, currently known strains could still harbor undisclosed signal not apparent in absence of one or both of the parental lineages, and should therefore be re-examined as new strains are discovered. Finally, a large part of the area where TBEV is known to be present has not been sampled, which means that our knowledge of its genetic variation is largely incomplete.

In conclusion, we have inferred the conditions required for a recombination signal to be deemed reliable. As such, we are providing a switch that will help reveal genuine recombinations lurking in the dark. Locating the cat in the room will require more simulations.

## Supporting Information

S1 File Figs A-J and Tables A-J. Fig A, False positive rates in simulations without recombination for 100 replicates for the FE-, W- and S- subtypes. Fig B, True and false positive rates in simulations with recombination for 100 replicates for the recombination events 1–2 in the FE-subtype at four recombination fragment sizes (200, 1000, 2000 and 3000 bp). Fig C, True and false positive rates in simulations with recombination for 100 replicates for the recombination event 3–4 in the FE-subtype. Fig D, True and false positive rates in simulations with recombination for 100 replicates for the recombination events 1–2 in the S-subtype. Fig E, True and false positive rates in simulations with recombination for 100 replicates for the recombination events 3 in the S-subtype. Fig F, True and false positive rates in simulations with recombination for 100 replicates for the recombination events 1–2 in the W-subtype. Fig G, True and false positive rates in simulations with recombination for 100 replicates for the recombination events 3 in the W-subtype. Fig H, Relationship between strength of detection and q for different detection methods. Fig I, MCC tree for E-gene sequences inferred from BEAST. Fig J, Phylogenetic analyses of recombination events from the empirical dataset described in Table 3. Table A, Genbank accession, location, sampling date and subtype identity of the full-length sequences used to compile the ALN alignments. Table B, Results of the RDP4 analyses carried on one hundred simulated datasets with recombination at detection *P-*value of 0.05 and without requiring agreement between multiple methods. Table C, Results of the RDP4 analyses carried on one hundred simulated datasets with recombination at detection *P-*value of 1,0E-6 and without requiring agreement between multiple methods. Table D, Results of the RDP4 analyses carried on one hundred simulated datasets with recombination at detection *P-*value of 1.0E-9 and without requiring agreement between multiple methods. Table E, Results of the RDP4 analyses carried on one hundred simulated datasets with recombination at detection *P-*value of 0.05 and requiring the agreement between at least two methods. Table F, Results of the RDP4 analyses carried on one hundred simulated datasets with recombination at detection *P-*value of 1.0E-6 and requiring the agreement between at least two methods. Table G, Results of the RDP4 analyses carried on one hundred simulated datasets with recombination at detection *P-*value of 1.0E-9 and requiring the agreement between at least two methods. Table H, Results of the RDP4 analyses carried on one hundred simulated datasets with recombination at detection *P-*value of 0.05 and requiring the agreement between at least three methods. Table I, Results of the RDP4 analyses carried on one hundred simulated datasets with recombination at detection *P-*value of 1.0E-6 and requiring the agreement between at least three methods. Table J, Results of the RDP4 analyses carried on one hundred simulated datasets with recombination at detection *P-*value of 1.0E-9 and requiring the agreement between at least three methods.
(PDF)
