# Supplementary material for: Revisiting Recombination Signal in the Tick-Borne Encephalitis Virus: A Simulation Approach
Source: PLoS One. 2016 Oct 19;11(10):e0164435. doi: 10.1371/journal.pone.0164435 (PMC5070875; doi:10.1371/journal.pone.0164435)
Supplement: S1 File — Fig A, False positive rates in simulations without recombination for 100 replicates for the FE-, W- and S- subtypes. Fig B, True and false positive rates in simulations with recombination for 100 replicates for the recombination events 1–2 in the FE-subtype at four recombination fragment sizes (200, 1000, 2000 and 3000 bp). Fig C, True and false positive rates in simulations with recombination for 100 replicates for the recombination event 3–4 in the FE-subtype. Fig D, True and false positive rates in simulations with recombination for 100 replicates for the recombination events 1–2 in the S-subtype. Fig E, True and false positive rates in simulations with recombination for 100 replicates for the recombination events 3 in the S-subtype. Fig F, True and false positive rates in simulations with recombination for 100 replicates for the recombination events 1–2 in the W-subtype. Fig G, True and false positive rates in simulations with recombination for 100 replicates for the recombination events 3 in the W-subtype. Fig H, Relationship between strength of detection and q for different detection methods. Fig I, MCC tree for E-gene sequences inferred from BEAST. Fig J, Phylogenetic analyses of recombination events from the empirical dataset described in Table 3. Table A, Genbank accession, location, sampling date and subtype identity of the full-length sequences used to compile the ALN alignments. Table B, Results of the RDP4 analyses carried on one hundred simulated datasets with recombination at detection P-value of 0.05 and without requiring agreement between multiple methods. Table C, Results of the RDP4 analyses carried on one hundred simulated datasets with recombination at detection P-value of 1,0E-6 and without requiring agreement between multiple methods. Table D, Results of the RDP4 analyses carried on one hundred simulated datasets with recombination at detection P-value of 1.0E-9 and without requiring agreement between multiple methods. Table E, Results of the [file pone.0164435.s001.pdf]

Research manuscript for *PlosONE*

TITLE – Revisiting recombination signal in the Tick-Borne Encephalitis Virus: a simulation approach

Short title: Study of recombination in TBEV with *in silico* simulations

Yann J. K. Bertrand,[1]#, Magnus Johansson,[2,3,4] Peter Norberg[5]

[1]*Science and Historical Investigations of Evolution Laboratory of Duba, Czech Rep.*

[2]*School of Medical Sciences Örebro University, Örebro, Sweden*

[3]*School of Natural Science, Technology & Environmental Studies, Södertörn University, Huddinge, Sweden*

[4]*iRiSC - Inflammatory Response and Infection Susceptibility Centre, Faculty of Medicine and Health, Örebro University, Örebro, Sweden*

[5]*Department of Clinical Microbiology, Sahlgrenska University, Gothenburg, Sweden*

#Address correspondence to Yann J. K. Bertrand, yjk_bertrand@ybertrand.org.

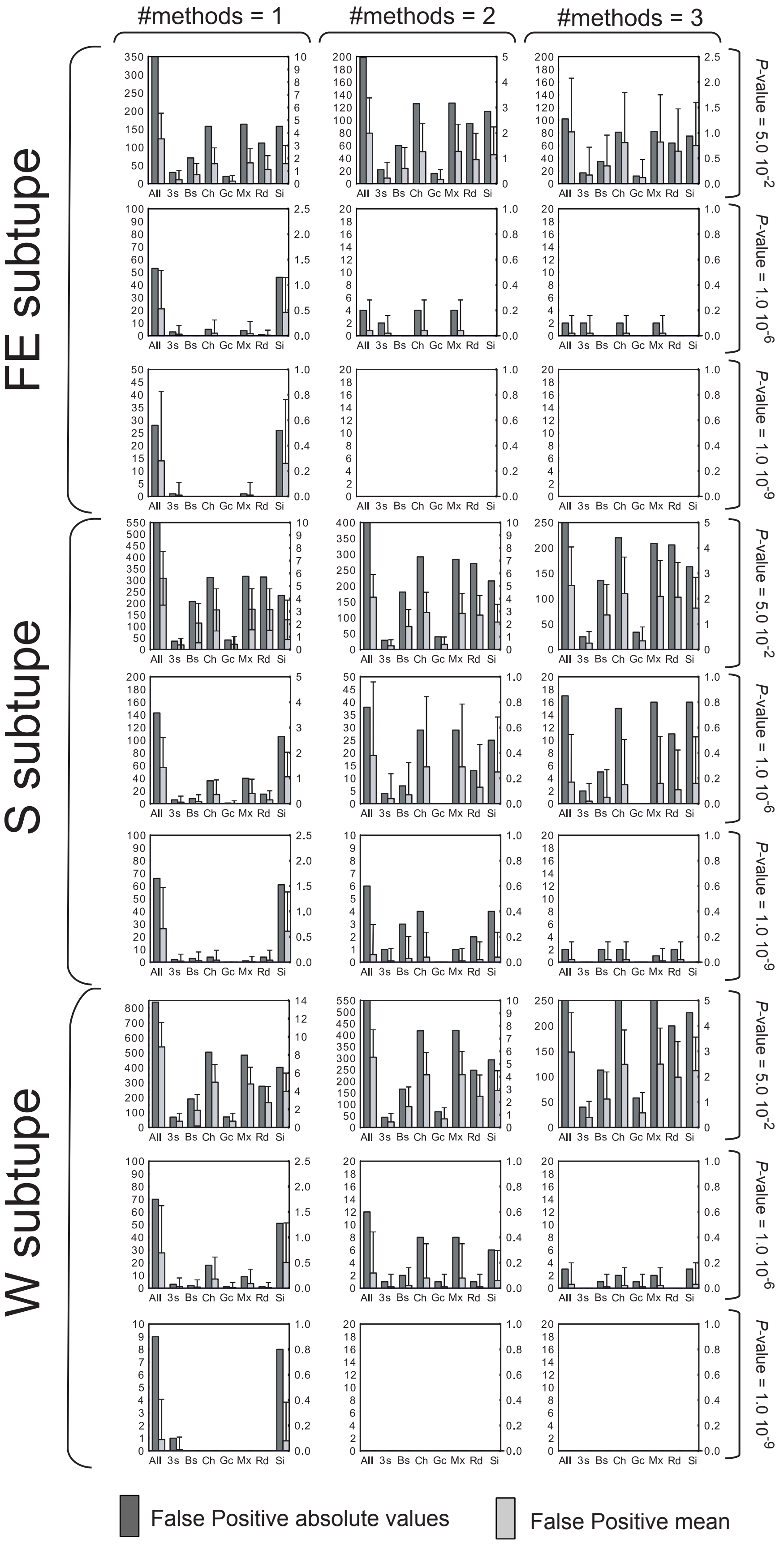

Figure A. False positive rates in simulations without recombination for 100 replicates for the FE-, W- and S-subtypes. Three detection P-values (0.05, 1E-6 and 1E-9) were tested, in combination with three screening strategies: reporting all events (#methods=1), requiring the agreement between two (#methods=2) or three (#methods=3) methods. Scale on the left hand side of a bar chart indicates the number of false positive records; scale on the right hand side indicates the mean per replicate. The standard deviation is represented for the mean value. "All" corresponds to the total detected events when all methods are considered. For false detection rates reported from individual method, the abbreviations are as follows: 3seq (3s), BootScan (Bs), Chimaera (Ch), GENECONV (Gc), MaxChi (Mx), RDP (Rd) and SiScan (Si).

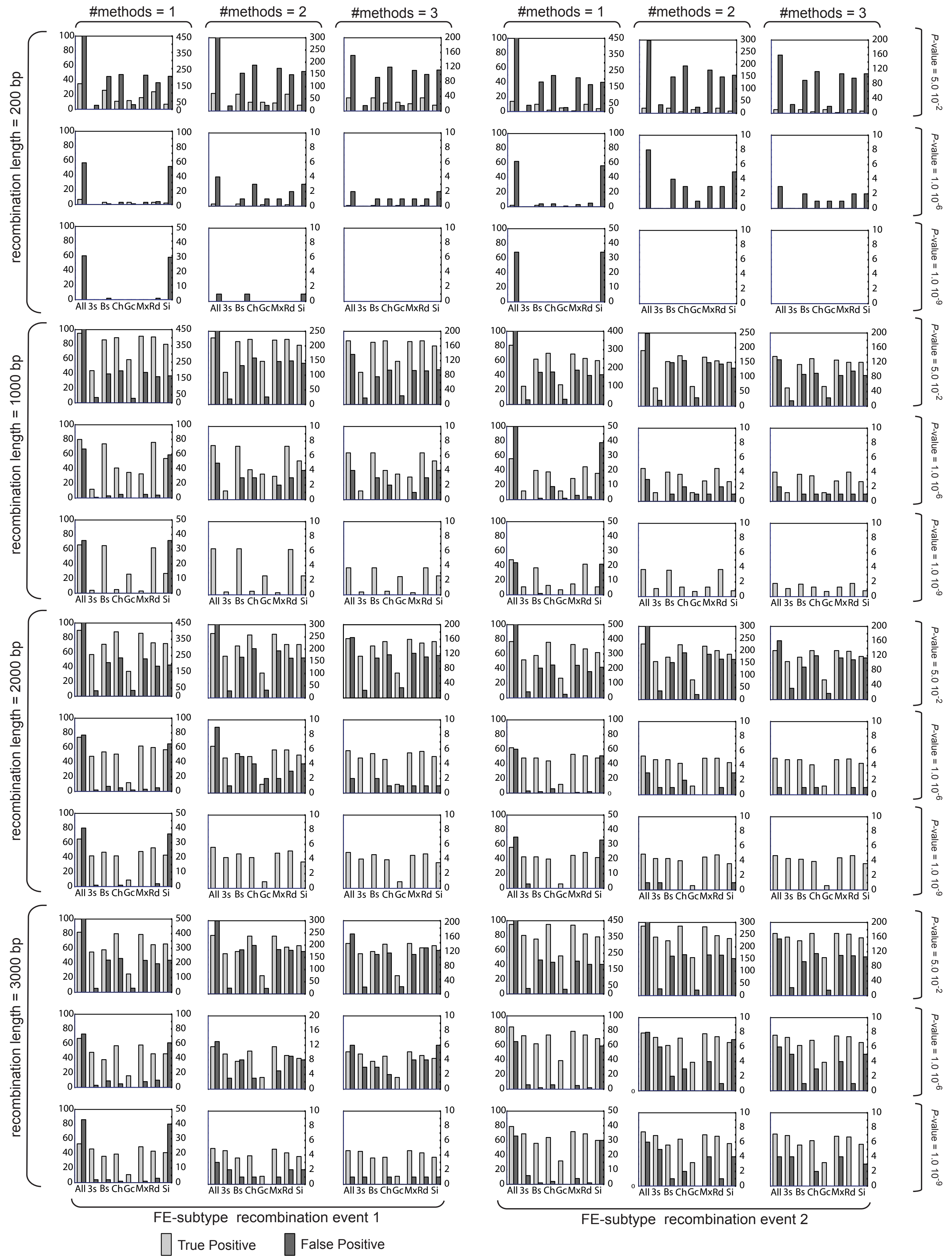

Figure B. True and false positive rates in simulations with recombination for 100 replicates for the recombination event 1-2 in the FE-subtype. See Figure A in S1 file for detailed descriptions of this figure.

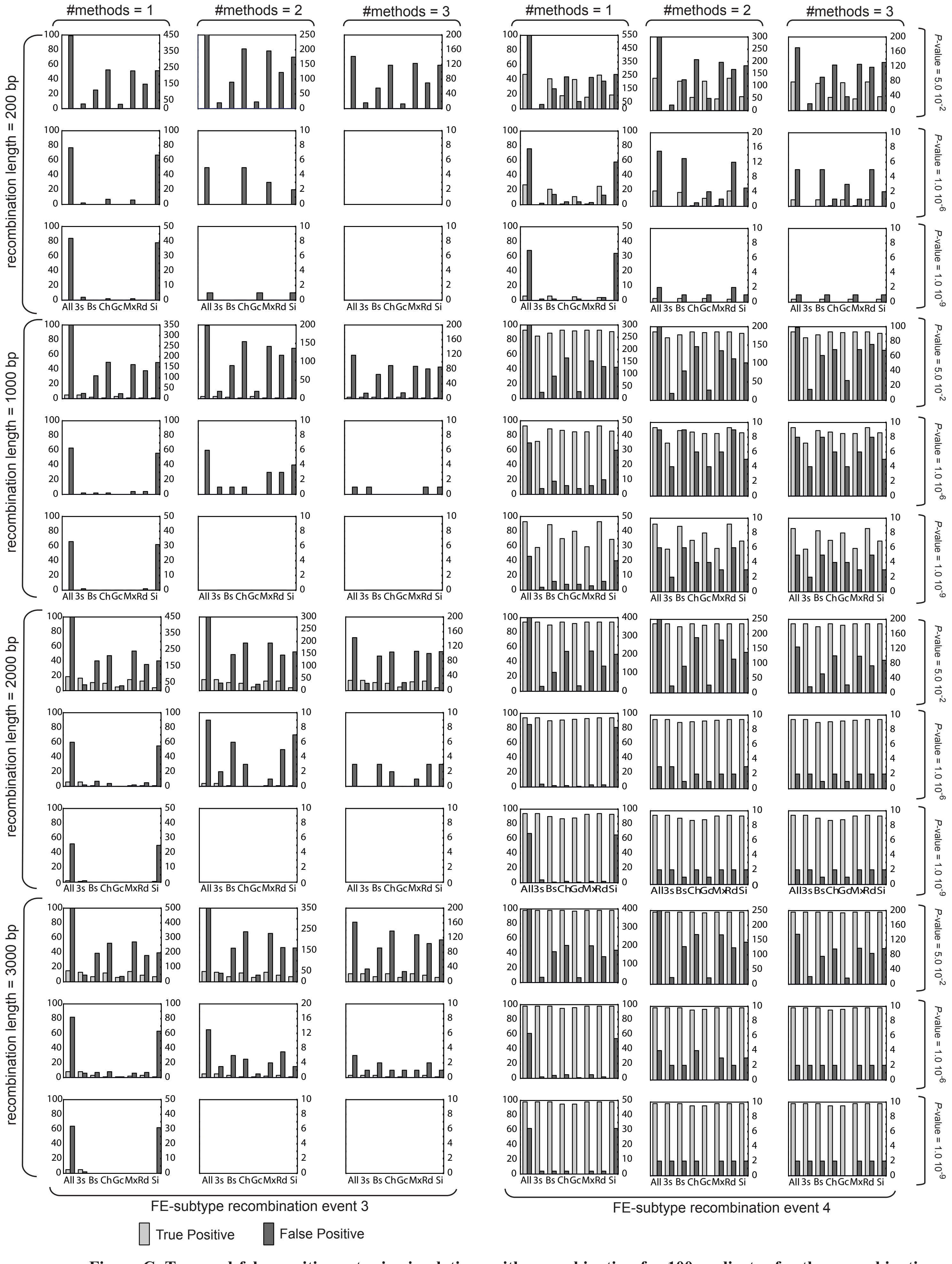

Figure C. True and false positive rates in simulations with recombination for 100 replicates for the recombination event 3-4 in the FE-subtype. See Figure A in S1 file for detailed descriptions of this figure.

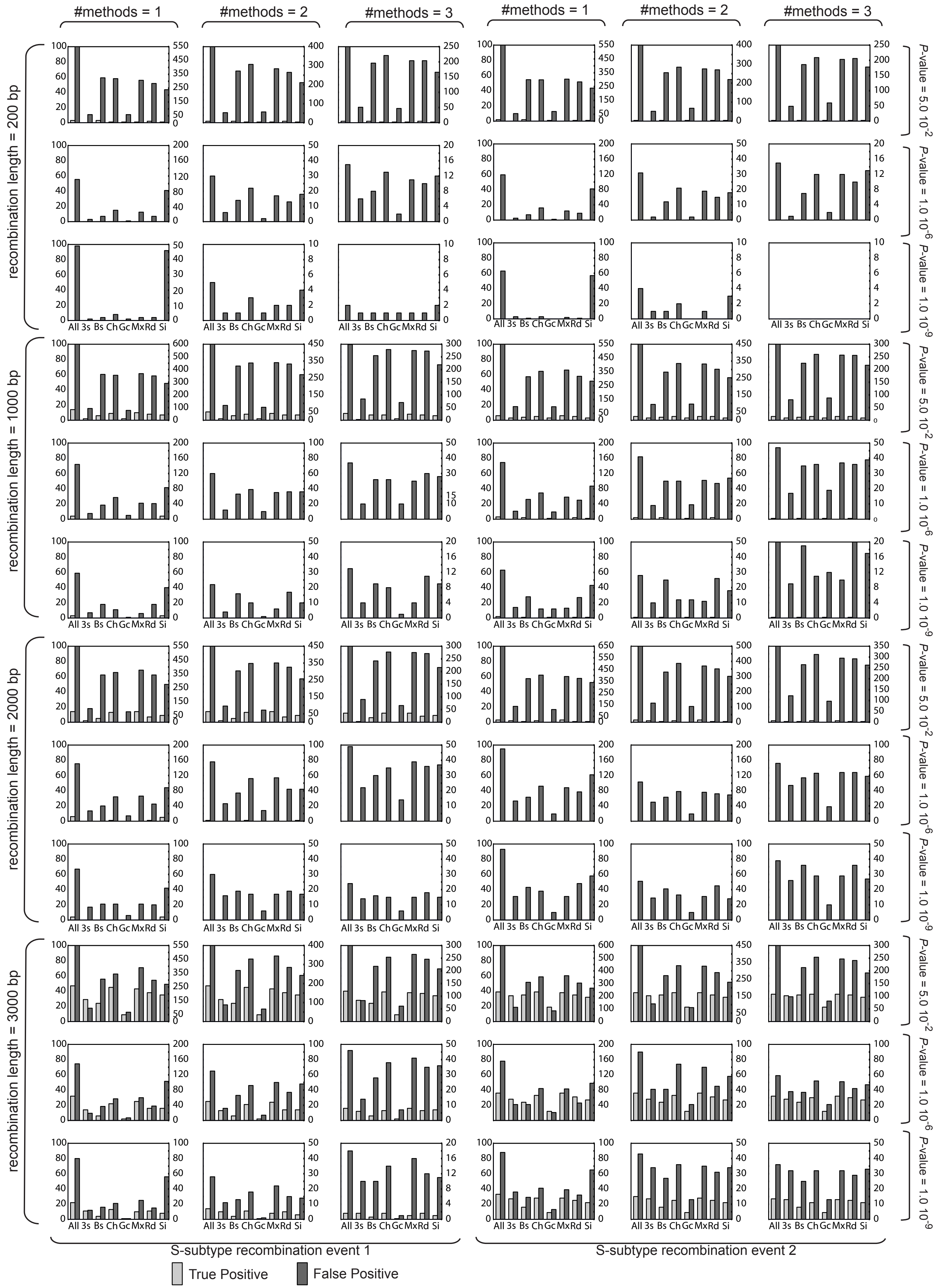

Figure D. True and false positive rates in simulations with recombination for 100 replicates for the recombination events 1-2 in the S-subtype. See Figure A in S1 file for detailed descriptions of this figure.

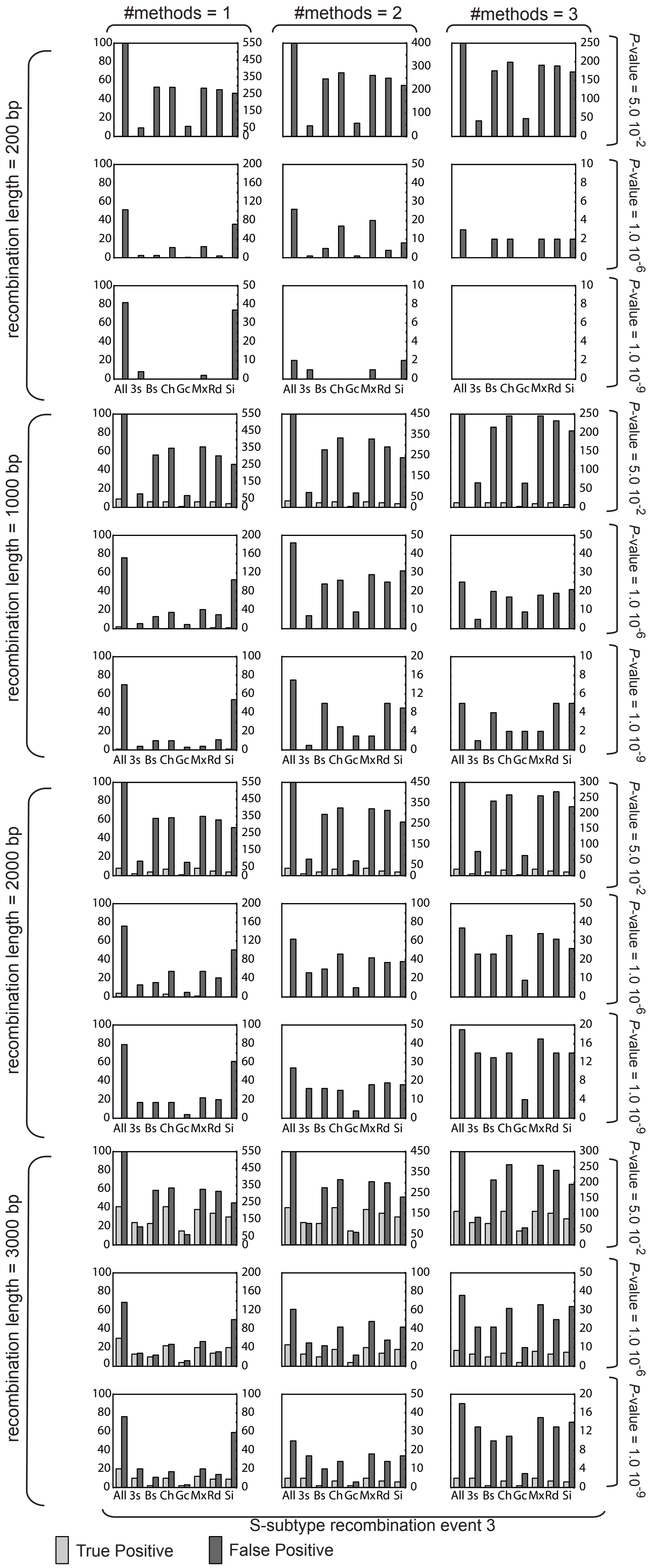

Figure E. True and false positive rates in simulations without recombination for 100 replicates for the recombination events 3 in the S-subtype. See Figure A in S1 file for detailed descriptions of this figure.

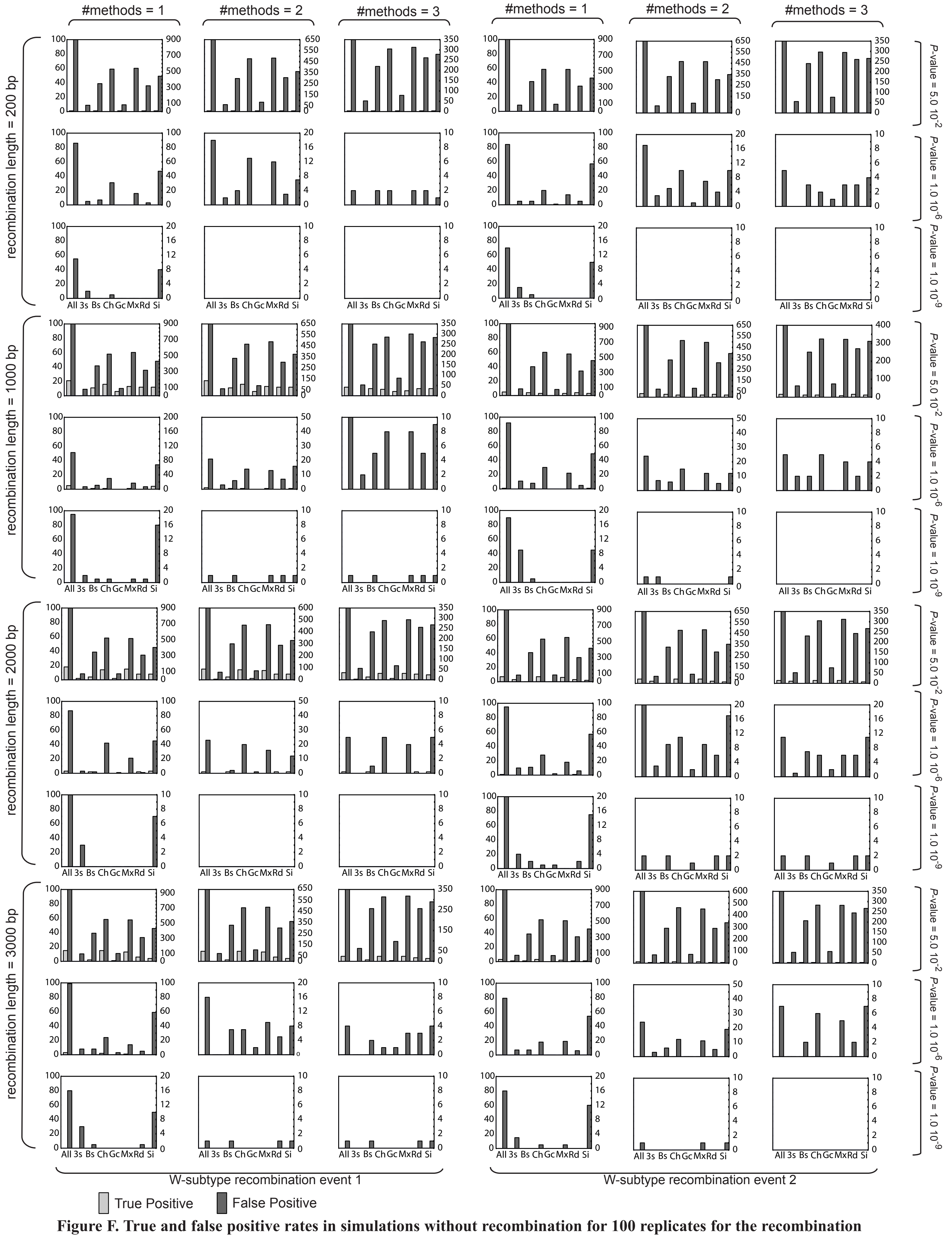

Figure F. True and false positive rates in simulations without recombination for 100 replicates for the recombination events 1-2 in the W-subtype. See Figure A in S1 file for detailed descriptions of this figure.

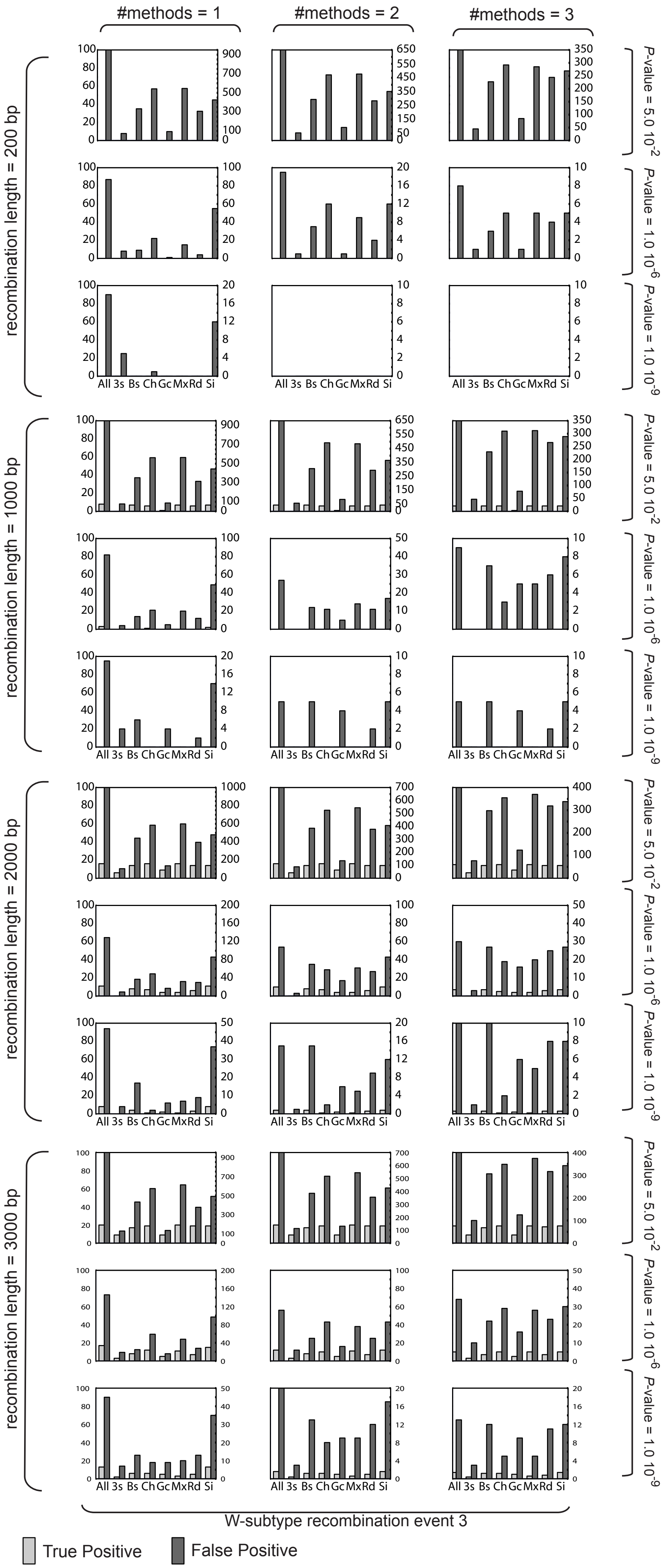

Figure G. True and false positive rates in simulations without recombination for 100 replicates for the recombination events 3 in the W-subtype. See Figure A in S1 file for detailed descriptions of this figure.

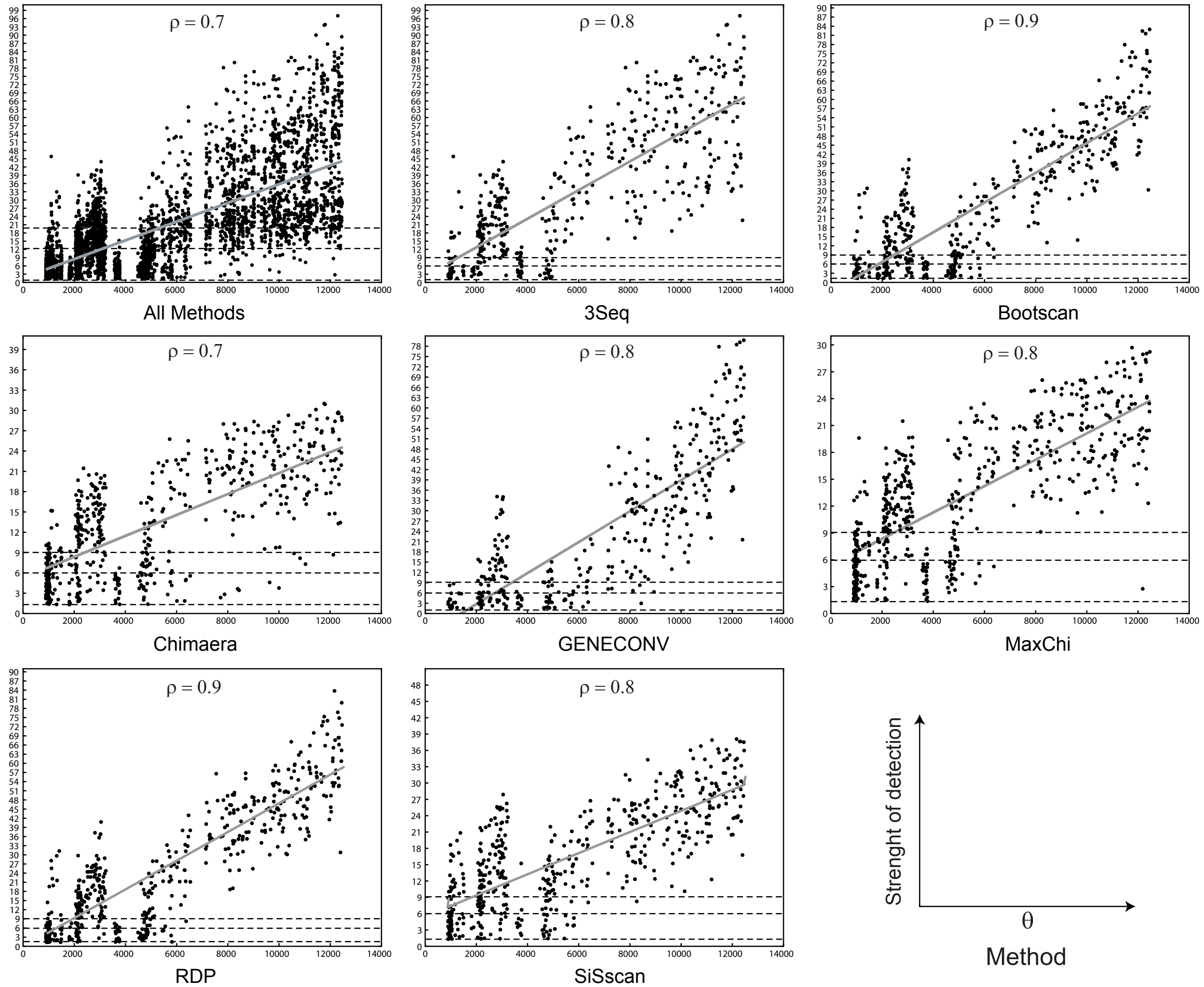

Figure H. Relationship between strength of detection and Θ for different detection methods. Data points correspond to positive detections of a genuine recombination event involving 2000 and 3000 bp fragments in any of the three subtypes for each detection method. The strength of the detection is measured as the log10 transformation of the inverse (f(x) =1/x) of the associated *P*-values. Each panel displays the regression line and the value of the Pearson product-moment correlation coefficient (ρ) associated with the data. The dashed lines represent detection threshold of *P*-values at 0.05, 1E-6 and 1E-9 (from lower to upper line in each panel).

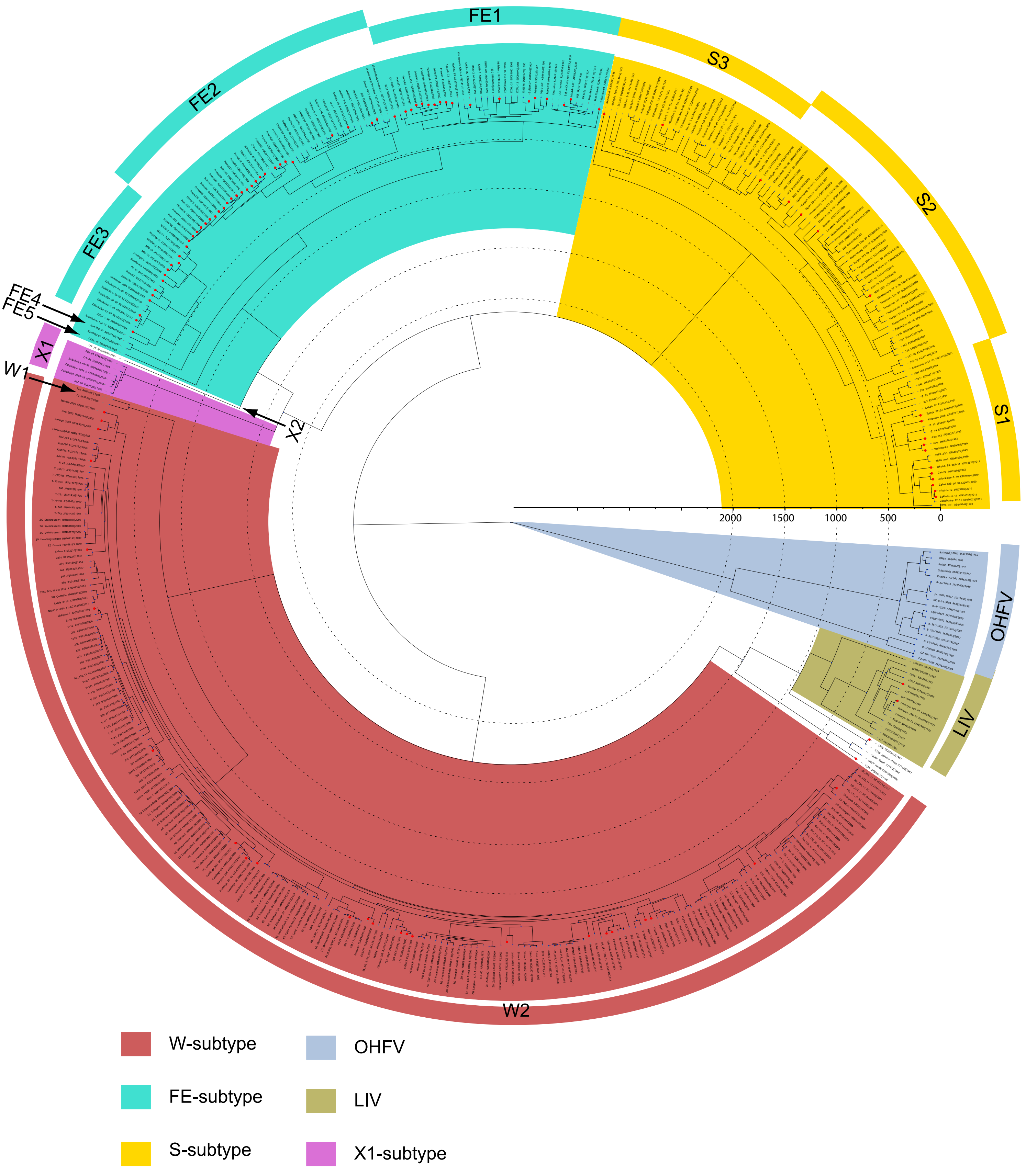

Figure I. MCC tree for E-gene sequences inferred from BEAST. The data set contains all E-gene sequences with collection date for W-, S-, FE-TBEV, OHFV, LIV, TSEV and SSEV available in GenBank at the time of March 2015. The scale is in years before present. X1 and X2 are provisional names for two clades of central Russian strains that are closely related to previously known FE-subtype strains, which cluster into three FE-crown clades. Red dot on terminal leaves indicate the strains for which a full genome sequence has been screened for recombination signal in the current study. Three lineages with no available full-genome sequences, W1, FE4 and FE5, have deeper divergences than most strains in their respective subtypes. They should therefore harbor sufficient sequence differences to detect ancient recombination event with another lineage of their own subtype, if such event has taken place in history. Sequence name is followed by GenBank accession and collection date.

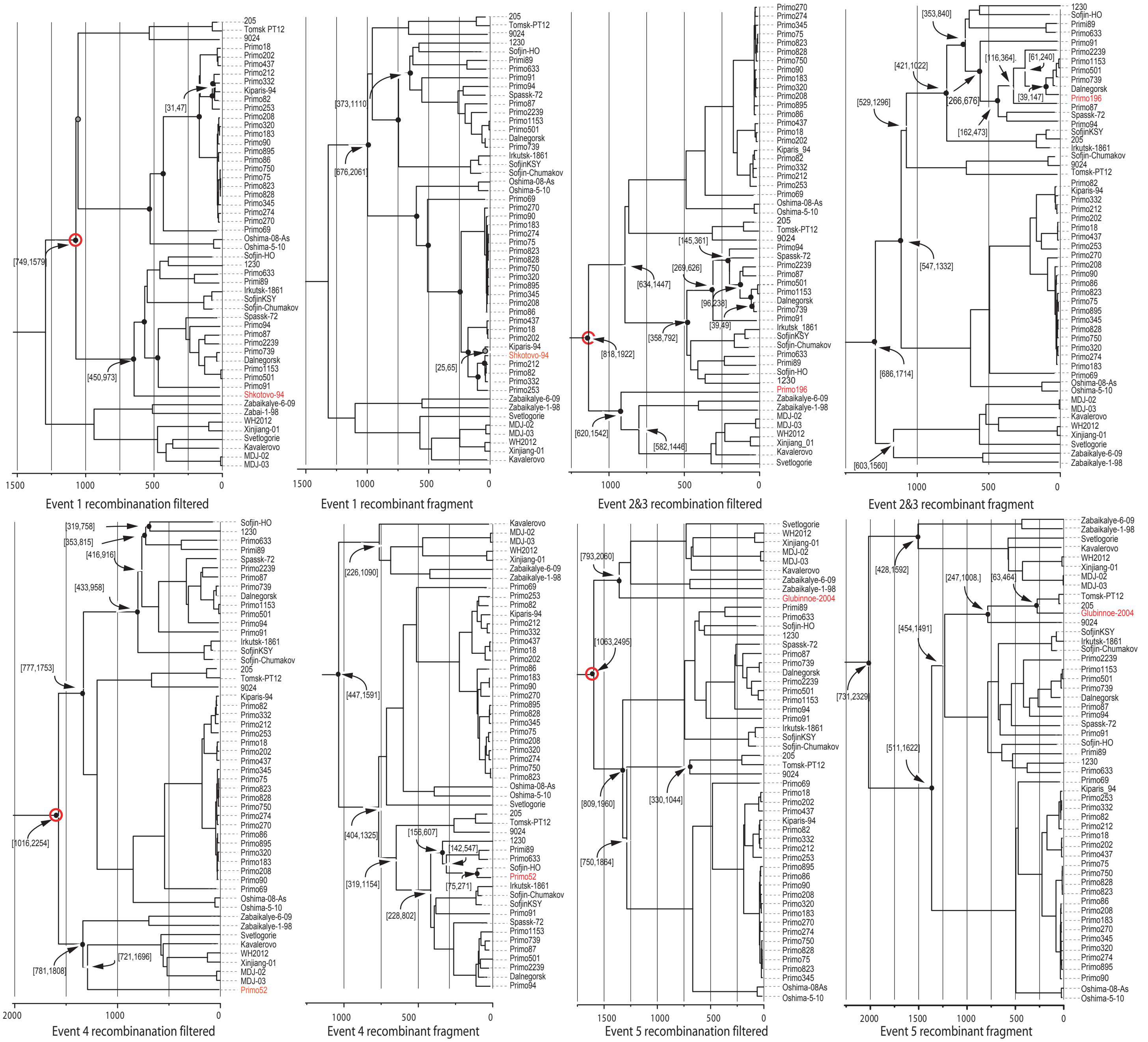

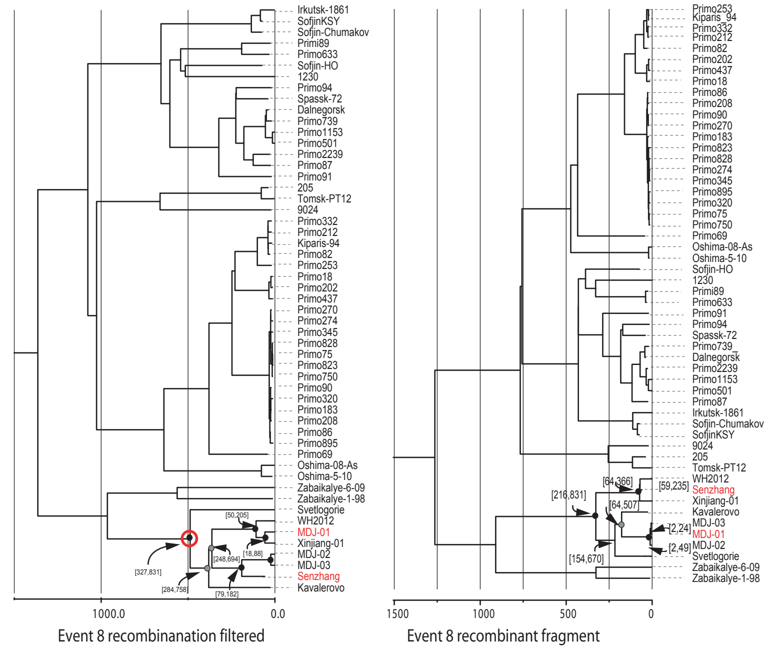

Figure J. Phylogenetic analyses of recombination events. MCC trees were inferred in BEAST from modified ALN2 alignments. For each putative recombination event, ALN2 was stripped from all identified putative recombinant sequences except for the strain at test. The resulting alignment was partitioned between non-recombining (left) and recombining regions (right) and trees were constructed separately for each partition. The name of the recombination strains is indicated in red. For a few selected nodes, relevant to the recombination analysis, the 95% HPD for the inferred dating is indicated along with the branch support (PP) that is represented by colouring the nodes in different shades of grey (black: PP >= 0.95, grey: 0.75 < PP < 0.95, uncoloured PP < 0.75).

Table A. Genbank accession, locations, sampling dates and subtype identity of the full-length sequences used to compile the ALN alignments.

Strain	Genbank Acc.	Geographical origin	Date of isolation	Clade
263	U27491	Czech Rep.: Temelin	1987	W-
2689	JQ693478	Russia: Novosibirsk	6/1/2010	S-
9024	KF880803	Russia: Amur region	1990	FE-
Aina	AF091006	Russia: Irkutsk region	1963	S-
AS33	GQ266392	Germany: Amberg	2005	W-
Cht-22	JN003208	Russia: Chita region	2002	S-
Cht-653	JN003207	Russia: Chita region	1995	S-
Dalnegorsk	FJ402886	Russia: Dalnegorsk	1973	FE-
Est3476	DQ393776	Estonia	2000	W-
Est54	DQ393773	Estonia	2000	S-
Glubinnoe	DQ862460	Russia: Primorsky krai	2004	FE-
Hypr	X75286	Czech Rep.: Brno	1953	W-
Irkutsk-12	JN003209	Russia: Irkutsk	2010	S-
Irkutsk-1861	JN003205	Russia: Siberia	2008	FE-
Joutseno	GU183381	Finland	1960	W-
K23	AF091010	Germany: Karlsruhe	1975	W-
Kavalerovo	FJ402885	Russia: Kavalerovsky	7/20/1985	FE-
Kiparis-94	JQ825146	Russia: Nadezhdinsky,Kiparisovo	4/17/1994	FE-
Kolarovo-2008	FJ968751	Russia: Kolarovo	5/1/2008	S-
KrM 213	EU276110	South Korea	2006	W-
KrM 93	EU276109	South Korea	2006	W-
Kumlinge 25-03	GU183379	Finland	2003	W-
Kumlinge A52	GU183380	Finland	1952	W-
Latvia 1-96	AJ415565	Latvia	2001	S-
Ljubljana I	AF091012	Slovenia: Ljubljana	1993	W-
Mandal-2009	KF991107	Norway: Mandal	2009	W-
MDJ-02	JF316707	China: Mudanjiang	6/1/2010	FE-
MDJ-03	JF316708	China: Mudanjiang	6/17/2010	FE-
MucAr M14/10	JF274481	Mongolia	7/1/2010	S-
Neudoerfl	U27495	Austria: Neudoerfl	1971	W-
Oshima 08-As	AB753012	Japan: Hokkaido, Hokuto	2008	FE-
Primorye-1153	HQ901366	Russia: Primorsky krai	2009	FE-
Primorye-18	GQ228395	Russia: Vladivostok	4/13/1997	FE-
Primorye-183	JQ825153	Russia: Nadezhdinsk	5/7/1991	FE-
Primorye-196	JQ825155	Russia: Lazurnaya	5/18/2000	FE-
Primorye-202	JQ825157	Russia: Nadezhdinsky, Solovey Klyuch	8/7/1997	FE-
Primorye-208	JQ825158	Russia: Primorsky krai	5/20/1991	FE-
Primorye-212	EU816450	Russia: Vladivostok	1991	FE-
Primorye-253	EU816451	Russia: Nadezhdinsky, Solovey Kluch	1991	FE-
Primorye-270	EU816452	Russia: Nadezhdinsky, Mirny	1991	FE-
Primorye-274	JQ825159	Russia: Nadezhdinsk	5/18/1999	FE-
Primorye-320	JQ825160	Russia: Shkotovo	5/24/1999	FE-
Primorye-332	AY169390	Russia: Nadezhdinsky	6/19/1991	FE-
Primorye-345	JQ825161	Russia: Vladivostok	5/25/1999	FE-
Primorye-437	JQ825162	Russia: Lazurnaya	6/1/1999	FE-
Primorye-501	HQ901367	Russia: Primorsky krai	2010	FE-
Primorye-52	JQ825154	Russia: Shkotovsky, Anisimovka	4/19/1999	FE-
Primorye-69	EU816453	Russia: Ussuriysk	1969	FE-
Primorye-739	JQ825156	Russia: within the city of Vladivostok	5/26/1992	FE-
Primorye-75	JQ825152	Russia: Nadezhdinsky	4/26/1999	FE-
Primorye-750	JQ825163	Russia: Nadezhdinsky, Kiparisovo	8/5/1998	FE-
Primorye-82	JQ825148	Russia: Vladivostok	5/28/1982	FE-
Primorye-823	JQ825164	Russia: Krasnoarmeysky	7/24/2000	FE-
Primorye-828	JQ825144	Russia: Chernigovka	8/25/1998	FE-
Primorye-86	EU816455	Russia: Kirovsky	1986	FE-
Primorye-87	JQ825149	Russia: Kavalerovsky	7/7/1987	FE-
Primorye-895	JQ825145	Primorsky kray, Far-East, Russia,	8/7/2000	FE-
Primorye-90	FJ997899	Russia: Primorsky krai	6/18/1990	FE-
Primorye-91	JQ825150	Russia: Primorsky krai	6/4/1991	FE-
Primorye-94	EU816454	Russia: Nadezhdinsky	6/16/1905	FE-
Saringe-2009	KF991106	Sweden: Saringe	2009	W-
Senzhang	AY174188	China: Heilongjiang Province	1953	FE-
Sofjin-Chumakov	KC806252	Russia: Primorsky krai	1937	FE-
Sofjin-HO	AB062064	Russia: Primorsky krai	1937	FE-

Sofjin-KSY	JF819648	Russia: Primorsky Krai	1937	FE-
Spassk-72	JQ825151	Russia: Spassk	5/4/1972	FE-
SSEV	DQ235152	Spain: el Pais Vasco	1987	SSEV
Svetlogorie	GU121642	Russia	7/28/2008	FE-
Torö-2003	DQ401140	Sweden: Torö	2003	W-
Vasilchenko	M97659	Russia	1969	S-
Xinjiang-01	JX534167	China	5/1/2012	FE-
Zabaikalye 68B-00	KF826914	Russia	2009	S-
Zabaikalye-1-98	JX968560	Russia	5/27/1998	FE-
Zabaikalye-30-00	KC422667	Russia: Zabaikalsky region	2000	FE-
Zausaev	AF527415	Russia: Tomsk	1985	S-

Table B. Results of the RDP4 analyses carried on one hundred simulated datasets with recombination at detection *P*-value of 0.05 and without requiring agreement between multiple methods.

	event	Recombination length	Method															
			All		3Seq		Bootscan		Chimaera		GENECONV		Maxchi		RDP		SiSscan	
			TP	FP	TP	FP	TP	FP	TP	FP	TP	FP	TP	FP	TP	FP	TP	FP
Subtype FE	1	200	18	5.6 (2.73)	0	0.26 (0.5)	8	1.4 (1.22)	2	2.55 (1.62)	5	0.26 (0.5)	4	2.43 (1.75)	11	1.88 (1.35)	6	2.6 (1.91)
		1000	84	5.02 (2.69)	10	0.29 (0.6)	62	1.27 (1.15)	77	2.1 (1.72)	20	0.28 (0.55)	71	2.27 (1.94)	67	1.78 (1.4)	65	2.18 (1.99)
		2000	87	5.0 (2.22)	51	0.18 (0.5)	68	1.05 (1.1)	84	2.38 (1.69)	18	0.23 (0.47)	85	2.37 (1.69)	73	1.64 (1.29)	75	2.12 (1.46)
	2	200	27	5.63 (3.17)	1	0.23 (0.58)	17	1.26 (1.14)	4	2.59 (1.92)	20	0.28 (0.49)	4	2.52 (1.8)	18	1.7 (1.35)	10	2.39 (2.59)
		1000	98	5.22 (2.3)	32	0.31 (0.5)	83	1.1 (0.96)	89	2.6 (1.68)	48	0.33 (0.57)	89	2.35 (1.44)	96	1.65 (1.19)	75	2.14 (1.81)
		2000	99	4.98 (2.98)	89	0.42 (0.66)	50	0.72 (1.16)	99	2.27 (1.57)	57	0.31 (0.52)	99	2.23 (1.64)	98	1.58 (1.37)	93	2.12 (2.4)
	3	200	0	4.92 (2.32)	0	0.33 (0.57)	0	1.07 (1.13)	0	2.22 (1.32)	0	0.22 (0.48)	0	2.29 (1.24)	0	1.46 (1.22)	0	2.07 (1.41)
		1000	5	4.34 (2.01)	4	0.26 (0.48)	4	1.16 (1.05)	1	1.92 (1.38)	3	0.23 (0.44)	2	1.83 (1.47)	1	1.39 (1.19)	1	1.77 (1.72)
		2000	2	4.58 (2.32)	2	0.33 (0.51)	2	1.14 (1.1)	1	2.08 (1.52)	2	0.13 (0.33)	2	2.1 (1.49)	1	1.35 (1.22)	2	1.96 (1.43)
	4	200	82	4.77 (2.57)	3	0.36 (0.64)	38	0.64 (0.92)	28	2.15 (1.51)	65	0.39 (0.61)	31	2.15 (1.63)	80	1.41 (1.2)	32	2.17 (1.93)
		1000	100	3.78 (2.01)	90	0.24 (0.47)	75	0.45 (0.74)	100	1.65 (1.19)	99	0.17 (0.43)	100	1.66 (1.27)	100	1.28 (1.17)	100	1.78 (1.58)
		2000	100	3.82 (2.32)	100	0.26 (0.52)	69	0.54 (0.83)	100	1.56 (1.29)	100	0.3 (0.52)	100	1.51 (1.28)	100	1.22 (1.13)	100	1.63 (1.72)
Subtype S	1	200	22	6.47 (2.3)	2	0.43 (0.6)	15	3.24 (1.73)	10	3.41 (1.64)	9	0.64 (0.76)	10	3.48 (1.66)	13	3.35 (1.65)	7	2.68 (1.69)
		1000	75	6.06 (2.46)	20	0.69 (0.94)	43	1.93 (1.34)	67	3.41 (1.82)	33	0.7 (0.73)	70	3.53 (1.82)	58	3.21 (1.51)	54	2.85 (1.51)
		2000	83	6.25 (2.64)	50	0.74 (1.0)	56	2.02 (1.62)	81	3.31 (1.82)	28	0.63 (0.75)	80	3.38 (1.79)	66	3.34 (1.74)	69	2.7 (1.65)
	2	200	25	6.48 (2.59)	1	0.61 (0.83)	9	2.26 (1.74)	7	3.56 (1.79)	14	0.61 (0.73)	8	3.72 (1.83)	17	3.41 (1.95)	7	2.76 (1.81)
		2000	85	6.88 (2.69)	74	0.99 (1.05)	80	2.2 (1.89)	85	3.96 (1.78)	42	0.76 (0.87)	85	4.11 (1.82)	85	3.86 (1.97)	83	3.4 (1.77)
		1000	85	6.47 (2.33)	46	0.8 (1.05)	77	1.99 (1.72)	84	3.5 (1.71)	49	0.83 (0.97)	85	3.69 (1.74)	82	3.67 (1.98)	75	3.17 (1.54)
	3	200	12	6.1 (2.5)	2	0.51 (0.78)	5	2.08 (1.56)	1	3.31 (1.71)	4	0.6 (0.75)	2	3.25 (1.77)	3	3.18 (1.8)	5	2.64 (1.64)
		1000	48	5.89 (2.78)	13	0.5 (0.77)	25	1.68 (1.48)	42	3.46 (1.84)	17	0.62 (0.82)	40	3.46 (1.89)	36	3.01 (1.8)	35	2.52 (1.63)
		2000	65	6.48 (2.5)	35	0.65 (0.88)	40	1.66 (1.45)	63	3.69 (1.72)	25	0.6 (0.83)	64	3.71 (1.62)	53	3.38 (1.84)	48	2.95 (1.64)
Subtype W	1	200	1	8.04 (2.57)	1	0.9 (1.0)	0	2.04 (1.57)	1	4.68 (1.7)	0	0.79 (0.83)	1	4.56 (2.05)	0	2.61 (1.48)	0	3.95 (2.06)
		1000	9	8.26 (2.64)	0	0.75 (0.93)	4	2.21 (1.61)	4	4.65 (1.99)	3	0.69 (0.9)	7	4.78 (2.08)	4	2.71 (1.69)	7	4.16 (2.06)
		2000	16	8.36 (2.48)	2	1.08 (1.19)	3	2.22 (1.58)	15	5.14 (1.87)	1	0.94 (1.0)	10	5.15 (1.79)	7	2.86 (1.67)	10	4.13 (1.97)
	2	200	0	8.08 (2.63)	0	0.93 (1.06)	0	2.13 (1.7)	0	4.52 (1.8)	0	0.9 (0.94)	0	4.78 (2.13)	0	2.5 (1.63)	0	4.0 (1.84)
		1000	2	8.3 (2.66)	0	1.1 (1.12)	0	2.19 (1.45)	1	4.75 (1.83)	0	0.8 (0.95)	2	4.6 (1.94)	0	2.87 (1.49)	0	4.26 (1.86)
		2000	4	8.11 (2.4)	1	1.12 (1.25)	1	2.17 (1.57)	2	4.5 (1.63)	0	0.76 (0.83)	3	4.8 (1.91)	2	2.81 (1.38)	2	4.08 (1.94)
	3	200	0	8.32 (2.21)	0	1.11 (1.21)	0	1.82 (1.24)	0	4.66 (1.99)	0	0.72 (0.75)	0	5.15 (2.1)	0	2.82 (1.56)	0	3.82 (2.02)
		1000	1	8.62 (2.67)	0	0.91 (1.12)	0	2.5 (1.8)	0	5.0 (2.1)	0	1.01 (1.19)	0	5.26 (2.22)	1	3.13 (1.83)	1	4.27 (1.99)
		2000	3	8.93 (2.57)	0	1.21 (1.28)	1	2.22 (1.49)	3	5.21 (1.93)	1	0.82 (0.96)	2	5.34 (1.83)	2	3.1 (1.66)	2	4.28 (2.09)

TP and FP stand for true and false positive detections respectively. True positive detections are reported as the total number of accurately identified recombination events across all simulations, whereas the values indicated for false detection are the mean value per replicate along with the standard deviation. The column “All” reports the TP and FP values across all detection methods.

Table C. Results of the RDP4 analyses carried on one hundred simulated datasets with recombination at detection *P*-value of 1.0E-6 and without requiring agreement between multiple methods.

	event	Recombination length	Method															
			All		3Seq		Bootscan		Chimaera		GENECONV		Maxchi		RDP		SiSscan	
			TP	FP	TP	FP	TP	FP	TP	FP	TP	FP	TP	FP	TP	FP	TP	FP
Subtype FE	1	200	7	0.57 (0.86)	0	0.0 (0.0)	3	0.01 (0.1)	0	0.03 (0.17)	3	0.01 (0.1)	0	0.03 (0.17)	3	0.04 (0.2)	2	0.52 (0.74)
		1000	56	0.5 (0.73)	12	0.0 (0.0)	40	0.01 (0.1)	38	0.09 (0.32)	12	0.01 (0.1)	29	0.03 (0.17)	45	0.02 (0.14)	36	0.39 (0.65)
		2000	62	0.6 (0.72)	48	0.03 (0.17)	48	0.02 (0.14)	44	0.06 (0.24)	12	0.0 (0.0)	53	0.01 (0.1)	51	0.02 (0.14)	48	0.51 (0.66)
		3000	67	0.73 (0.93)	48	0.03 (0.17)	38	0.09 (0.38)	57	0.05 (0.22)	16	0.0 (0.0)	58	0.08 (0.31)	46	0.1 (0.33)	46	0.61 (0.77)
	2	200	2	0.62 (0.77)	0	0.0 (0.0)	2	0.04 (0.2)	0	0.04 (0.2)	0	0.01 (0.1)	0	0.03 (0.17)	0	0.05 (0.22)	0	0.56 (0.77)
		1000	80	0.67 (0.81)	12	0.01 (0.1)	74	0.03 (0.17)	41	0.05 (0.26)	35	0.0 (0.0)	33	0.05 (0.22)	76	0.04 (0.2)	54	0.59 (0.74)
		2000	74	0.77 (0.88)	48	0.02 (0.14)	54	0.07 (0.26)	51	0.05 (0.22)	12	0.02 (0.14)	62	0.03 (0.17)	60	0.05 (0.22)	57	0.65 (0.83)
		3000	85	0.65 (0.8)	73	0.06 (0.24)	62	0.02 (0.14)	74	0.06 (0.28)	39	0.0 (0.0)	79	0.05 (0.22)	74	0.02 (0.14)	69	0.59 (0.75)
	3	200	0	0.77 (0.97)	0	0.02 (0.14)	0	0.0 (0.0)	0	0.07 (0.26)	0	0.0 (0.0)	0	0.06 (0.24)	0	0.0 (0.0)	0	0.67 (0.86)
		1000	0	0.63 (0.81)	0	0.02 (0.14)	0	0.02 (0.14)	0	0.02 (0.14)	0	0.0 (0.0)	0	0.04 (0.2)	0	0.04 (0.24)	0	0.56 (0.79)
		2000	6	0.6 (0.8)	6	0.02 (0.14)	1	0.07 (0.29)	0	0.04 (0.2)	0	0.0 (0.0)	1	0.02 (0.14)	1	0.05 (0.26)	1	0.55 (0.77)
		3000	8	0.82 (0.99)	8	0.06 (0.24)	3	0.07 (0.26)	1	0.08 (0.27)	1	0.01 (0.1)	2	0.06 (0.28)	3	0.07 (0.26)	1	0.63 (0.82)
	4	200	27	0.76 (0.91)	0	0.02 (0.14)	21	0.14 (0.37)	1	0.04 (0.2)	11	0.04 (0.2)	1	0.03 (0.17)	25	0.13 (0.36)	0	0.58 (0.81)
		1000	93	0.35 (0.61)	72	0.04 (0.2)	89	0.09 (0.29)	87	0.06 (0.24)	85	0.04 (0.2)	85	0.06 (0.24)	93	0.1 (0.33)	86	0.3 (0.54)
		2000	94	0.85 (1.67)	94	0.04 (0.2)	90	0.02 (0.14)	91	0.02 (0.14)	92	0.01 (0.1)	93	0.03 (0.17)	94	0.03 (0.17)	94	0.81 (1.66)
		3000	98	0.61 (0.8)	98	0.02 (0.14)	98	0.04 (0.2)	95	0.05 (0.22)	96	0.01 (0.1)	98	0.05 (0.3)	98	0.02 (0.14)	98	0.54 (0.73)
Subtype S	1	200	0	1.11 (1.06)	0	0.06 (0.24)	0	0.14 (0.35)	0	0.3 (0.56)	0	0.02 (0.14)	0	0.25 (0.46)	0	0.14 (0.35)	0	0.82 (0.93)
		1000	4	1.44 (1.21)	0	0.15 (0.41)	0	0.37 (0.58)	0	0.57 (0.8)	0	0.1 (0.3)	0	0.42 (0.65)	0	0.41 (0.6)	4	0.83 (0.84)
		2000	6	1.51 (1.2)	0	0.27 (0.61)	0	0.4 (0.6)	1	0.64 (0.78)	0	0.14 (0.35)	0	0.66 (0.72)	1	0.45 (0.61)	5	0.88 (1.01)
		3000	32	1.49 (1.2)	14	0.19 (0.42)	6	0.37 (0.61)	22	0.57 (0.72)	2	0.07 (0.26)	25	0.6 (0.72)	16	0.38 (0.63)	16	1.03 (1.0)
	2	200	0	1.19 (1.2)	0	0.05 (0.41)	0	0.14 (0.35)	0	0.32 (0.65)	0	0.02 (0.14)	0	0.24 (0.51)	0	0.18 (0.41)	0	0.82 (0.9)
		1000	3	1.49 (1.16)	0	0.21 (0.45)	2	0.52 (0.64)	0	0.69 (0.7)	1	0.19 (0.42)	0	0.58 (0.71)	2	0.5 (0.62)	1	0.87 (0.98)
		2000	0	1.9 (1.24)	0	0.53 (0.57)	0	0.63 (0.59)	0	0.92 (0.7)	0	0.19 (0.39)	0	0.88 (0.72)	0	0.77 (0.63)	0	1.22 (1.09)
		3000	36	1.56 (1.23)	28	0.42 (0.57)	24	0.42 (0.53)	33	0.84 (0.81)	12	0.21 (0.43)	36	0.83 (0.87)	31	0.46 (0.57)	27	0.98 (0.91)
	3	200	0	0.05 (0.22)	0	0.05 (0.22)	0	0.22 (0.46)	0	0.01 (0.1)	0	0.24 (0.49)	0	0.04 (0.2)	0	0.72 (0.94)	0	0.05 (0.22)
		1000	0	0.11 (0.34)	0	0.26 (0.44)	0	0.35 (0.55)	0	0.09 (0.29)	0	0.41 (0.72)	1	0.3 (0.5)	1	1.05 (1.12)	0	0.11 (0.34)
		2000	0	0.26 (0.44)	0	0.31 (0.46)	3	0.55 (0.67)	0	0.1 (0.3)	1	0.55 (0.78)	0	0.41 (0.55)	0	1.01 (0.93)	0	0.26 (0.44)
		3000	13	0.28 (0.53)	10	0.24 (0.45)	22	0.47 (0.7)	4	0.12 (0.35)	20	0.53 (0.77)	14	0.31 (0.52)	20	1.0 (0.99)	13	0.28 (0.53)
Subtype W	1	200	0	0.86 (1.06)	0	0.05 (0.26)	0	0.07 (0.26)	0	0.31 (0.61)	0	0.0 (0.0)	0	0.16 (0.42)	0	0.03 (0.17)	0	0.47 (0.79)
		1000	5	1.02 (1.07)	0	0.07 (0.29)	1	0.11 (0.37)	1	0.3 (0.64)	0	0.0 (0.0)	1	0.17 (0.43)	0	0.07 (0.26)	4	0.68 (0.84)
		2000	3	0.87 (0.97)	0	0.03 (0.17)	2	0.02 (0.14)	0	0.42 (0.71)	0	0.01 (0.1)	0	0.21 (0.48)	2	0.01 (0.1)	3	0.45 (0.71)
		3000	3	0.99 (0.98)	0	0.08 (0.31)	0	0.08 (0.31)	2	0.24 (0.51)	0	0.03 (0.17)	1	0.14 (0.4)	0	0.05 (0.26)	0	0.59 (0.76)
	2	200	0	0.84 (0.97)	0	0.05 (0.26)	0	0.05 (0.26)	0	0.2 (0.51)	0	0.01 (0.1)	0	0.14 (0.37)	0	0.05 (0.22)	0	0.57 (0.72)
		1000	1	0.92 (1.13)	0	0.11 (0.44)	0	0.08 (0.27)	0	0.3 (0.54)	0	0.0 (0.0)	0	0.22 (0.52)	0	0.05 (0.22)	1	0.49 (0.73)
		2000	1	0.95 (1.06)	0	0.1 (0.41)	0	0.11 (0.34)	0	0.28 (0.57)	0	0.02 (0.2)	0	0.18 (0.54)	1	0.06 (0.28)	0	0.57 (0.7)
		3000	0	0.79 (1.0)	0	0.07 (0.26)	0	0.07 (0.26)	0	0.18 (0.43)	0	0.0 (0.0)	0	0.19 (0.5)	0	0.06 (0.24)	0	0.54 (0.84)
	3	200	0	0.87 (0.98)	0	0.08 (0.34)	0	0.09 (0.29)	0	0.22 (0.5)	0	0.01 (0.1)	0	0.15 (0.36)	0	0.04 (0.2)	0	0.55 (0.79)

		1000	3	0.82 (0.86)	0	0.04 (0.2)	0	0.14 (0.35)	1	0.21 (0.48)	0	0.05 (0.22)	0	0.2 (0.45)	0	0.12 (0.32)	2	0.49 (0.7)
		2000	11	1.29 (1.13)	0	0.09 (0.32)	8	0.37 (0.54)	7	0.49 (0.7)	4	0.17 (0.38)	4	0.32 (0.6)	6	0.3 (0.48)	11	0.86 (0.86)
		3000	17	1.46 (1.04)	3	0.19 (0.44)	8	0.25 (0.46)	12	0.59 (0.69)	5	0.16 (0.39)	11	0.48 (0.62)	7	0.28 (0.45)	15	0.97 (0.97)

TP and FP stand for true and false positive detections respectively. True positive detections are reported as the total number of accurately identified recombination events across all simulations, whereas the values indicated for false detection are the mean value per replicate along with the standard deviation. The column “All” reports the TP and FP values across all detection methods.

Table D. Results of the RDP4 analyses carried on one hundred simulated datasets with recombination at detection *P*-value of 1.0E-9 and without requiring agreement between multiple methods.

	event	Recombination length	Method															
			All		3Seq		Bootscan		Chimaera		GENECONV		Maxchi		RDP		SiSscan	
			TP	FP	TP	FP	TP	FP	TP	FP	TP	FP	TP	FP	TP	FP	TP	FP
Subtype FE	1	200	0	0.3 (0.54)	0	0.0 (0.0)	0	0.01 (0.1)	0	0.0 (0.0)	0	0.0 (0.0)	0	0.0 (0.0)	0	0.01 (0.1)	0	0.29 (0.52)
		1000	48	0.22 (0.46)	11	0.0 (0.0)	37	0.01 (0.1)	13	0.0 (0.0)	7	0.0 (0.0)	15	0.0 (0.0)	42	0.0 (0.0)	11	0.21 (0.45)
		2000	56	0.35 (0.57)	43	0.03 (0.17)	43	0.0 (0.0)	40	0.0 (0.0)	6	0.0 (0.0)	45	0.0 (0.0)	49	0.0 (0.0)	42	0.33 (0.53)
		3000	53	0.43 (0.65)	46	0.02 (0.14)	36	0.02 (0.14)	39	0.01 (0.1)	11	0.0 (0.0)	49	0.01 (0.1)	43	0.03 (0.17)	41	0.4 (0.65)
	2	200	0	0.34 (0.55)	0	0.0 (0.0)	0	0.0 (0.0)	0	0.0 (0.0)	0	0.0 (0.0)	0	0.0 (0.0)	0	0.0 (0.0)	0	0.34 (0.55)
		1000	66	0.36 (0.52)	4	0.0 (0.0)	65	0.0 (0.0)	5	0.0 (0.0)	26	0.0 (0.0)	3	0.0 (0.0)	62	0.0 (0.0)	27	0.36 (0.52)
		2000	65	0.4 (0.62)	42	0.01 (0.1)	47	0.0 (0.0)	42	0.01 (0.1)	9	0.0 (0.0)	48	0.0 (0.0)	53	0.02 (0.14)	43	0.36 (0.57)
		3000	79	0.33 (0.55)	69	0.06 (0.24)	56	0.01 (0.1)	64	0.02 (0.14)	32	0.0 (0.0)	72	0.04 (0.2)	69	0.01 (0.1)	60	0.3 (0.54)
	3	200	0	0.42 (0.71)	0	0.02 (0.14)	0	0.0 (0.0)	0	0.01 (0.1)	0	0.0 (0.0)	0	0.01 (0.1)	0	0.0 (0.0)	0	0.39 (0.65)
		1000	0	0.33 (0.51)	0	0.01 (0.1)	0	0.0 (0.0)	0	0.0 (0.0)	0	0.0 (0.0)	0	0.0 (0.0)	0	0.01 (0.1)	0	0.31 (0.5)
		2000	2	0.26 (0.48)	1	0.01 (0.1)	0	0.0 (0.0)	0	0.0 (0.0)	0	0.0 (0.0)	0	0.0 (0.0)	0	0.0 (0.0)	1	0.25 (0.48)
		3000	5	0.32 (0.56)	5	0.01 (0.1)	0	0.0 (0.0)	0	0.0 (0.0)	0	0.0 (0.0)	0	0.0 (0.0)	0	0.0 (0.0)	0	0.31 (0.56)
	4	200	6	0.34 (0.72)	0	0.01 (0.1)	6	0.01 (0.1)	0	0.0 (0.0)	5	0.01 (0.1)	0	0.0 (0.0)	4	0.02 (0.14)	0	0.32 (0.71)
		1000	93	0.23 (0.47)	58	0.02 (0.14)	89	0.06 (0.24)	70	0.04 (0.2)	80	0.04 (0.2)	59	0.03 (0.17)	93	0.06 (0.24)	69	0.2 (0.45)
		2000	94	0.67 (1.5)	94	0.04 (0.2)	90	0.01 (0.1)	87	0.02 (0.14)	88	0.01 (0.1)	93	0.02 (0.14)	94	0.02 (0.14)	93	0.65 (1.5)
		3000	98	0.31 (0.56)	98	0.02 (0.14)	98	0.02 (0.14)	95	0.02 (0.14)	95	0.0 (0.0)	98	0.02 (0.14)	98	0.02 (0.14)	98	0.31 (0.56)
Subtype S	1	200	0	0.49 (0.79)	0	0.01 (0.1)	0	0.02 (0.14)	0	0.04 (0.2)	0	0.01 (0.1)	0	0.02 (0.14)	0	0.02 (0.14)	0	0.46 (0.79)
		1000	3	0.59 (0.72)	0	0.07 (0.29)	0	0.18 (0.41)	0	0.11 (0.4)	0	0.01 (0.1)	0	0.06 (0.24)	0	0.18 (0.41)	3	0.4 (0.6)
		2000	4	0.67 (0.87)	0	0.17 (0.45)	0	0.21 (0.48)	0	0.21 (0.45)	0	0.06 (0.24)	0	0.21 (0.45)	0	0.2 (0.45)	4	0.42 (0.75)
		3000	22	0.8 (0.88)	11	0.12 (0.32)	4	0.16 (0.39)	13	0.21 (0.45)	1	0.01 (0.1)	10	0.25 (0.48)	11	0.15 (0.36)	8	0.56 (0.78)
	2	200	0	0.63 (0.96)	0	0.03 (0.3)	0	0.01 (0.1)	0	0.03 (0.17)	0	0.0 (0.0)	0	0.02 (0.14)	0	0.01 (0.1)	0	0.57 (0.84)
		1000	2	0.63 (0.8)	0	0.14 (0.37)	1	0.28 (0.49)	0	0.12 (0.32)	1	0.12 (0.35)	0	0.13 (0.39)	1	0.27 (0.47)	1	0.43 (0.65)
		2000	0	0.93 (0.84)	0	0.31 (0.46)	0	0.43 (0.51)	0	0.38 (0.49)	0	0.1 (0.3)	0	0.31 (0.46)	0	0.48 (0.52)	0	0.58 (0.87)
		3000	33	0.88 (0.82)	27	0.36 (0.52)	16	0.29 (0.48)	28	0.41 (0.55)	9	0.13 (0.36)	28	0.39 (0.51)	25	0.32 (0.49)	22	0.65 (0.68)
	3	200	0	0.41 (0.71)	0	0.04 (0.2)	0	0.0 (0.0)	0	0.0 (0.0)	0	0.0 (0.0)	0	0.02 (0.14)	0	0.0 (0.0)	0	0.37 (0.7)
		1000	1	0.7 (0.91)	0	0.04 (0.24)	0	0.1 (0.3)	0	0.1 (0.33)	0	0.03 (0.17)	0	0.04 (0.2)	0	0.11 (0.31)	1	0.54 (0.77)
		2000	0	0.79 (0.8)	0	0.17 (0.38)	0	0.17 (0.38)	0	0.17 (0.38)	0	0.04 (0.2)	0	0.22 (0.48)	0	0.2 (0.4)	0	0.61 (0.66)
		3000	20	0.76 (0.87)	10	0.2 (0.4)	2	0.11 (0.34)	10	0.17 (0.4)	2	0.03 (0.22)	12	0.2 (0.42)	9	0.14 (0.37)	9	0.59 (0.81)
Subtype W	1	200	0	0.11 (0.37)	0	0.02 (0.14)	0	0.0 (0.0)	0	0.01 (0.1)	0	0.0 (0.0)	0	0.0 (0.0)	0	0.0 (0.0)	0	0.08 (0.34)
		1000	0	0.19 (0.46)	0	0.02 (0.14)	0	0.01 (0.1)	0	0.01 (0.1)	0	0.0 (0.0)	0	0.01 (0.1)	0	0.01 (0.1)	0	0.16 (0.39)
		2000	0	0.1 (0.3)	0	0.03 (0.17)	0	0.0 (0.0)	0	0.0 (0.0)	0	0.0 (0.0)	0	0.0 (0.0)	0	0.0 (0.0)	0	0.07 (0.26)
		3000	0	0.16 (0.42)	0	0.06 (0.28)	0	0.01 (0.1)	0	0.0 (0.0)	0	0.0 (0.0)	0	0.0 (0.0)	0	0.01 (0.1)	0	0.1 (0.33)
	2	200	0	0.14 (0.4)	0	0.03 (0.22)	0	0.01 (0.1)	0	0.0 (0.0)	0	0.0 (0.0)	0	0.0 (0.0)	0	0.0 (0.0)	0	0.1 (0.33)
		1000	0	0.18 (0.48)	0	0.09 (0.4)	0	0.01 (0.1)	0	0.0 (0.0)	0	0.0 (0.0)	0	0.0 (0.0)	0	0.0 (0.0)	0	0.09 (0.29)
		2000	0	0.2 (0.51)	0	0.04 (0.2)	0	0.02 (0.14)	0	0.01 (0.1)	0	0.01 (0.1)	0	0.0 (0.0)	0	0.02 (0.14)	0	0.15 (0.41)
		3000	0	0.16 (0.39)	0	0.03 (0.17)	0	0.0 (0.0)	0	0.01 (0.1)	0	0.0 (0.0)	0	0.01 (0.1)	0	0.0 (0.0)	0	0.12 (0.32)
	3	200	0	0.18 (0.46)	0	0.05 (0.26)	0	0.0 (0.0)	0	0.01 (0.1)	0	0.0 (0.0)	0	0.0 (0.0)	0	0.0 (0.0)	0	0.12 (0.35)
		1000	0	0.19 (0.42)	0	0.04 (0.2)	0	0.06 (0.24)	0	0.0 (0.0)	0	0.04 (0.2)	0	0.0 (0.0)	0	0.02 (0.14)	0	0.14 (0.35)

		2000	8	0.47 (0.66)	0	0.04 (0.2)	4	0.17 (0.38)	1	0.02 (0.14)	2	0.06 (0.24)	1	0.07 (0.26)	3	0.09 (0.29)	8	0.37 (0.58)
		3000	13	0.45 (0.57)	2	0.07 (0.26)	6	0.13 (0.34)	6	0.09 (0.29)	5	0.09 (0.29)	3	0.1 (0.3)	5	0.13 (0.34)	13	0.35 (0.54)

TP and FP stand for true and false positive detections respectively. True positive detections are reported as the total number of accurately identified recombination events across all simulations, whereas the values indicated for false detection are the mean value per replicate along with the standard deviation. The column “All” reports the TP and FP values across all detection methods.

Table E. Results of the RDP4 analyses carried on one hundred simulated datasets with recombination at detection *P*-value of 0.05 and requiring the agreement between at least two methods.

	event	Recombination length	Method															
			All		3Seq		Bootscan		Chimaera		GENECONV		Maxchi		RDP		SiSscan	
			TP	FP	TP	FP	TP	FP	TP	FP	TP	FP	TP	FP	TP	FP	TP	FP
Subtype FE	1	200	24	3.06 (1.69)	0	0.21 (0.45)	23	1.55 (1.28)	12	1.88 (1.37)	12	0.23 (0.51)	11	1.75 (1.41)	23	1.49 (1.2)	8	1.62 (1.22)
		1000	76	2.76 (1.96)	25	0.2 (0.45)	61	1.49 (1.35)	69	1.56 (1.46)	27	0.29 (0.5)	67	1.49 (1.47)	62	1.44 (1.35)	60	1.3 (1.2)
		2000	76	3.23 (1.62)	52	0.36 (0.59)	58	1.52 (1.29)	75	1.92 (1.16)	27	0.21 (0.48)	73	1.86 (1.08)	67	1.66 (1.34)	62	1.65 (1.13)
		3000	80	3.39 (1.73)	55	0.24 (0.49)	58	1.81 (1.34)	79	1.99 (1.32)	25	0.23 (0.53)	79	1.82 (1.18)	64	1.79 (1.39)	66	1.74 (1.42)
	2	200	7	3.22 (1.87)	0	0.35 (0.64)	7	1.49 (1.08)	2	1.94 (1.57)	5	0.25 (0.43)	1	1.77 (1.47)	7	1.49 (1.26)	3	1.56 (1.29)
		1000	91	2.78 (1.47)	44	0.19 (0.42)	86	1.33 (1.04)	89	1.59 (1.21)	59	0.26 (0.56)	88	1.47 (1.2)	89	1.49 (1.05)	81	1.41 (1.06)
		2000	88	3.27 (1.67)	57	0.29 (0.52)	71	1.67 (1.24)	86	2.02 (1.49)	34	0.32 (0.55)	87	1.93 (1.39)	73	1.63 (1.23)	73	1.64 (1.22)
		3000	95	3.03 (1.79)	80	0.28 (0.51)	75	1.62 (1.3)	95	1.68 (1.36)	52	0.23 (0.49)	94	1.67 (1.38)	82	1.66 (1.47)	78	1.52 (1.06)
	3	200	0	3.0 (1.96)	0	0.2 (0.49)	0	0.9 (1.03)	0	2.03 (1.61)	0	0.23 (0.47)	0	1.96 (1.59)	0	1.23 (1.14)	0	1.75 (1.45)
		1000	3	2.38 (1.7)	3	0.2 (0.42)	2	0.9 (1.1)	1	1.55 (1.25)	3	0.2 (0.49)	1	1.42 (1.2)	1	1.18 (1.13)	1	1.37 (1.27)
		2000	15	3.14 (1.91)	15	0.31 (0.5)	11	1.47 (1.47)	10	1.94 (1.55)	5	0.26 (0.59)	13	1.94 (1.51)	13	1.45 (1.4)	4	1.58 (1.29)
		3000	14	3.55 (1.8)	13	0.41 (0.58)	7	1.6 (1.26)	12	2.38 (1.47)	6	0.32 (0.61)	13	2.3 (1.5)	9	1.63 (1.29)	7	1.61 (1.38)
	4	200	44	3.25 (1.71)	0	0.23 (0.49)	40	1.26 (1.05)	18	2.08 (1.37)	40	0.5 (0.66)	16	1.97 (1.37)	44	1.68 (1.3)	19	1.83 (1.29)
		1000	93	2.18 (1.48)	85	0.19 (0.48)	89	0.8 (0.94)	93	1.46 (1.26)	92	0.28 (0.51)	93	1.35 (1.17)	93	1.13 (1.1)	91	1.02 (0.99)
		2000	94	2.79 (1.98)	94	0.24 (0.47)	90	0.91 (1.07)	94	1.88 (1.49)	92	0.27 (0.65)	94	1.8 (1.28)	94	1.15 (1.05)	94	1.38 (1.67)
		3000	98	2.8 (1.79)	98	0.23 (0.47)	98	1.28 (1.12)	98	1.69 (1.4)	97	0.22 (0.41)	98	1.68 (1.4)	98	1.24 (1.13)	98	1.43 (1.19)
Subtype S	1	200	2	4.47 (1.84)	0	0.53 (0.73)	2	2.72 (1.69)	1	3.07 (1.58)	1	0.57 (0.71)	1	2.84 (1.57)	2	2.65 (1.49)	1	2.11 (1.3)
		1000	11	4.97 (2.09)	2	0.88 (1.07)	6	3.21 (1.73)	8	3.39 (1.64)	2	0.77 (0.8)	9	3.41 (1.82)	7	3.33 (1.65)	7	2.7 (1.59)
		2000	14	4.76 (2.15)	2	0.96 (1.09)	5	3.04 (1.69)	13	3.48 (1.89)	0	0.72 (0.84)	14	3.51 (1.85)	7	3.26 (1.67)	9	2.57 (1.46)
		3000	47	4.48 (2.11)	29	0.88 (0.95)	24	2.69 (1.57)	45	3.29 (1.75)	9	0.66 (0.78)	43	3.45 (1.89)	38	2.85 (1.55)	35	2.43 (1.58)
	2	200	1	4.36 (1.84)	0	0.52 (0.88)	1	2.55 (1.51)	0	2.84 (1.64)	1	0.68 (0.82)	0	2.75 (1.6)	1	2.7 (1.39)	1	2.19 (1.5)
		1000	5	4.61 (1.93)	3	0.94 (1.11)	4	2.85 (1.48)	5	3.36 (1.66)	3	0.96 (0.86)	5	3.33 (1.6)	5	3.02 (1.61)	3	2.52 (1.47)
		2000	3	5.3 (2.01)	2	1.26 (0.99)	1	3.3 (1.77)	2	3.87 (1.65)	0	1.04 (0.93)	3	3.7 (1.76)	1	3.51 (1.64)	1	3.02 (1.65)
		3000	38	4.63 (2.16)	34	1.05 (1.06)	35	2.72 (1.76)	38	3.31 (1.71)	19	0.83 (0.98)	38	3.28 (1.75)	35	2.9 (1.75)	32	2.33 (1.45)
	3	200	0	4.2 (2.07)	0	0.46 (0.79)	0	2.47 (1.67)	0	2.73 (1.8)	0	0.57 (0.79)	0	2.62 (1.73)	0	2.5 (1.57)	0	2.19 (1.59)
		1000	7	4.71 (2.03)	0	0.72 (0.87)	5	2.78 (1.53)	6	3.35 (1.77)	1	0.7 (0.74)	6	3.3 (1.72)	5	2.92 (1.65)	4	2.4 (1.49)
		2000	8	4.62 (1.67)	2	0.8 (0.84)	4	2.96 (1.5)	7	3.27 (1.47)	1	0.72 (0.74)	8	3.23 (1.42)	5	3.15 (1.45)	4	2.59 (1.48)
		3000	40	4.53 (2.25)	24	1.04 (1.26)	23	2.76 (1.54)	40	3.15 (1.89)	15	0.61 (0.76)	38	3.05 (1.98)	34	3.0 (1.65)	30	2.31 (1.32)
Subtype W	1	200	1	6.62 (2.13)	0	0.63 (0.93)	1	2.99 (1.61)	0	4.78 (1.81)	1	0.85 (0.88)	0	4.84 (1.93)	1	3.07 (1.61)	1	3.62 (1.93)
		1000	21	6.79 (2.61)	0	0.64 (0.88)	11	3.39 (1.92)	16	4.67 (2.24)	6	0.92 (0.96)	13	4.89 (2.3)	12	3.04 (1.9)	12	3.76 (1.99)
		2000	15	6.2 (2.22)	1	0.64 (0.79)	4	3.01 (1.83)	14	4.57 (1.92)	2	0.76 (0.9)	13	4.61 (1.81)	8	2.89 (1.68)	8	3.29 (1.73)
		3000	14	6.68 (2.54)	0	0.71 (0.82)	2	3.27 (1.75)	14	4.85 (2.12)	1	1.04 (0.97)	13	4.9 (2.25)	6	3.02 (1.65)	4	3.61 (1.97)
	2	200	0	6.62 (2.2)	0	0.65 (0.95)	0	3.3 (1.8)	0	4.66 (2.05)	0	0.89 (1.01)	0	4.65 (1.91)	0	3.02 (1.67)	0	3.48 (1.83)
		1000	5	7.0 (2.6)	0	0.73 (1.12)	4	3.37 (1.97)	3	5.13 (2.34)	0	0.81 (1.03)	3	4.96 (2.32)	4	3.12 (1.69)	3	3.95 (2.11)
		2000	6	6.71 (2.07)	3	0.64 (1.02)	0	3.28 (1.41)	6	4.8 (2.12)	0	0.83 (0.98)	6	4.85 (2.03)	3	2.84 (1.36)	2	3.54 (1.47)
		3000	2	6.46 (2.34)	1	0.7 (0.97)	1	2.92 (1.6)	2	4.64 (2.0)	0	0.74 (0.84)	2	4.53 (2.02)	1	2.91 (1.61)	1	3.38 (1.68)
	3	200	0	6.61 (2.56)	0	0.55 (0.88)	0	2.95 (1.63)	0	4.71 (2.39)	0	0.94 (0.9)	0	4.77 (2.18)	0	2.85 (1.67)	0	3.52 (1.87)
		1000	7	6.61 (2.31)	0	0.59 (0.93)	7	3.07 (1.86)	6	4.92 (1.89)	1	0.86 (0.84)	6	4.85 (1.8)	6	2.95 (1.85)	7	3.66 (1.93)

		2000	16	7.39 (2.55)	6	0.88 (0.98)	14	3.86 (2.09)	16	5.25 (2.06)	9	1.35 (1.28)	16	5.44 (2.18)	14	3.78 (2.07)	14	4.07 (1.83)
		3000	20	7.25 (2.39)	9	1.13 (1.07)	17	3.85 (1.86)	19	5.17 (2.02)	9	1.31 (1.16)	20	5.44 (2.08)	19	3.55 (1.77)	19	4.26 (2.17)

TP and FP stand for true and false positive detections respectively. True positive detections are reported as the total number of accurately identified recombination events across all simulations, whereas the values indicated for false detection are the mean value per replicate along with the standard deviation. The column “All” reports the TP and FP values across all detection methods.

Table F. Results of the RDP4 analyses carried on one hundred simulated datasets with recombination at detection *P*-value of 1.0E-6 and requiring the agreement between at least two methods.

	event	recombination length	Method															
			All		3Seq		Bootscan		Chimaera		GENECONV		Maxchi		RDP		SiSscan	
			TP	FP	TP	FP	TP	FP	TP	FP	TP	FP	TP	FP	TP	FP	TP	FP
Subtype FE	1	200	3	0.04 (0.2)	0	0.0 (0.0)	3	0.01 (0.1)	0	0.03 (0.17)	2	0.01 (0.1)	0	0.01 (0.1)	2	0.02 (0.14)	0	0.03 (0.17)
		1000	45	0.03 (0.17)	12	0.0 (0.0)	40	0.01 (0.1)	37	0.02 (0.14)	12	0.01 (0.1)	28	0.01 (0.1)	45	0.02 (0.14)	27	0.01 (0.1)
		2000	53	0.03 (0.17)	48	0.01 (0.1)	48	0.01 (0.1)	43	0.02 (0.14)	12	0.0 (0.0)	50	0.0 (0.0)	50	0.01 (0.1)	44	0.03 (0.17)
		3000	58	0.13 (0.36)	48	0.03 (0.17)	38	0.08 (0.31)	52	0.03 (0.17)	16	0.0 (0.0)	58	0.05 (0.22)	46	0.09 (0.32)	42	0.08 (0.27)
	2	200	0	0.08 (0.27)	0	0.0 (0.0)	0	0.04 (0.2)	0	0.03 (0.17)	0	0.01 (0.1)	0	0.03 (0.17)	0	0.03 (0.17)	0	0.05 (0.22)
		1000	74	0.05 (0.22)	12	0.0 (0.0)	73	0.03 (0.17)	41	0.03 (0.17)	35	0.0 (0.0)	32	0.02 (0.14)	73	0.03 (0.17)	53	0.04 (0.2)
		2000	64	0.09 (0.29)	48	0.01 (0.1)	54	0.05 (0.22)	50	0.04 (0.2)	12	0.02 (0.14)	59	0.02 (0.14)	59	0.03 (0.17)	52	0.04 (0.2)
		3000	79	0.08 (0.27)	73	0.06 (0.24)	62	0.02 (0.14)	72	0.03 (0.17)	39	0.0 (0.0)	78	0.04 (0.2)	74	0.01 (0.1)	66	0.07 (0.26)
	3	200	0	0.05 (0.22)	0	0.0 (0.0)	0	0.0 (0.0)	0	0.05 (0.22)	0	0.0 (0.0)	0	0.03 (0.17)	0	0.0 (0.0)	0	0.02 (0.14)
		1000	0	0.06 (0.24)	0	0.01 (0.1)	0	0.01 (0.1)	0	0.01 (0.1)	0	0.0 (0.0)	0	0.03 (0.17)	0	0.03 (0.17)	0	0.04 (0.2)
		2000	4	0.09 (0.32)	4	0.02 (0.14)	1	0.06 (0.28)	0	0.03 (0.17)	0	0.0 (0.0)	1	0.01 (0.1)	1	0.05 (0.26)	1	0.07 (0.29)
		3000	5	0.13 (0.36)	5	0.03 (0.17)	3	0.06 (0.24)	1	0.05 (0.22)	1	0.01 (0.1)	2	0.04 (0.2)	3	0.07 (0.26)	1	0.03 (0.17)
	4	200	21	0.15 (0.38)	0	0.0 (0.0)	19	0.13 (0.36)	1	0.01 (0.1)	11	0.04 (0.2)	1	0.02 (0.14)	21	0.12 (0.35)	0	0.05 (0.22)
		1000	93	0.09 (0.29)	72	0.04 (0.2)	89	0.09 (0.29)	87	0.06 (0.24)	85	0.04 (0.2)	85	0.06 (0.24)	93	0.09 (0.29)	86	0.05 (0.22)
		2000	94	0.03 (0.17)	94	0.03 (0.17)	90	0.01 (0.1)	91	0.02 (0.14)	92	0.01 (0.1)	93	0.02 (0.14)	94	0.02 (0.14)	94	0.03 (0.17)
		3000	98	0.04 (0.2)	98	0.02 (0.14)	98	0.02 (0.14)	95	0.04 (0.2)	96	0.0 (0.0)	98	0.03 (0.17)	98	0.02 (0.14)	98	0.03 (0.17)
Subtype S	1	200	0	0.3 (0.56)	0	0.06 (0.24)	0	0.14 (0.35)	0	0.22 (0.46)	0	0.02 (0.14)	0	0.17 (0.38)	0	0.13 (0.34)	0	0.18 (0.41)
		1000	0	0.6 (0.73)	0	0.12 (0.35)	0	0.33 (0.57)	0	0.39 (0.65)	0	0.1 (0.3)	0	0.35 (0.61)	0	0.36 (0.57)	0	0.36 (0.56)
		2000	1	0.78 (0.82)	0	0.23 (0.55)	0	0.37 (0.59)	1	0.56 (0.74)	0	0.14 (0.35)	0	0.57 (0.71)	0	0.42 (0.6)	1	0.42 (0.64)
		3000	25	0.65 (0.77)	13	0.16 (0.39)	6	0.33 (0.6)	21	0.46 (0.64)	2	0.07 (0.26)	24	0.5 (0.64)	14	0.37 (0.63)	14	0.48 (0.67)
	2	200	0	0.31 (0.59)	0	0.02 (0.2)	0	0.12 (0.32)	0	0.21 (0.48)	0	0.02 (0.14)	0	0.19 (0.42)	0	0.15 (0.36)	0	0.18 (0.46)
		1000	2	0.82 (0.8)	0	0.18 (0.41)	2	0.5 (0.64)	0	0.5 (0.64)	1	0.19 (0.42)	0	0.51 (0.66)	2	0.47 (0.59)	0	0.54 (0.73)
		2000	0	1.03 (0.73)	0	0.5 (0.56)	0	0.63 (0.59)	0	0.78 (0.66)	0	0.19 (0.39)	0	0.76 (0.65)	0	0.72 (0.63)	0	0.69 (0.66)
		3000	36	0.9 (0.81)	28	0.41 (0.55)	24	0.41 (0.53)	33	0.74 (0.73)	12	0.21 (0.43)	36	0.7 (0.75)	31	0.45 (0.55)	27	0.58 (0.64)
	3	200	0	0.26 (0.5)	0	0.01 (0.1)	0	0.05 (0.22)	0	0.17 (0.43)	0	0.01 (0.1)	0	0.2 (0.45)	0	0.04 (0.2)	0	0.08 (0.27)
		1000	0	0.46 (0.67)	0	0.07 (0.26)	0	0.24 (0.43)	0	0.26 (0.5)	0	0.09 (0.29)	0	0.29 (0.57)	0	0.25 (0.46)	0	0.31 (0.58)
		2000	0	0.62 (0.7)	0	0.26 (0.44)	0	0.3 (0.46)	0	0.46 (0.62)	0	0.1 (0.3)	0	0.42 (0.59)	0	0.37 (0.52)	0	0.38 (0.54)
		3000	23	0.61 (0.86)	13	0.25 (0.52)	10	0.22 (0.44)	18	0.42 (0.7)	4	0.12 (0.35)	20	0.48 (0.74)	14	0.28 (0.49)	18	0.42 (0.62)
Subtype W	1	200	0	0.18 (0.48)	0	0.02 (0.14)	0	0.04 (0.2)	0	0.13 (0.42)	0	0.0 (0.0)	0	0.12 (0.38)	0	0.03 (0.17)	0	0.07 (0.29)
		1000	2	0.21 (0.55)	0	0.03 (0.17)	1	0.06 (0.28)	1	0.14 (0.51)	0	0.0 (0.0)	1	0.13 (0.36)	0	0.07 (0.26)	1	0.16 (0.46)
		2000	2	0.23 (0.49)	0	0.0 (0.0)	2	0.02 (0.14)	0	0.2 (0.47)	0	0.01 (0.1)	0	0.16 (0.39)	2	0.0 (0.0)	2	0.12 (0.35)
		3000	0	0.16 (0.42)	0	0.0 (0.0)	0	0.07 (0.29)	0	0.07 (0.29)	0	0.02 (0.14)	0	0.09 (0.32)	0	0.05 (0.26)	0	0.08 (0.27)
	2	200	0	0.17 (0.47)	0	0.03 (0.17)	0	0.05 (0.26)	0	0.1 (0.36)	0	0.01 (0.1)	0	0.07 (0.29)	0	0.04 (0.2)	0	0.1 (0.36)
		1000	0	0.24 (0.59)	0	0.07 (0.29)	0	0.06 (0.24)	0	0.15 (0.43)	0	0.0 (0.0)	0	0.12 (0.41)	0	0.05 (0.22)	0	0.12 (0.38)
		2000	0	0.2 (0.49)	0	0.03 (0.17)	0	0.09 (0.32)	0	0.11 (0.37)	0	0.02 (0.2)	0	0.09 (0.35)	0	0.06 (0.28)	0	0.17 (0.43)
		3000	0	0.24 (0.53)	0	0.03 (0.17)	0	0.06 (0.24)	0	0.12 (0.38)	0	0.0 (0.0)	0	0.11 (0.37)	0	0.05 (0.22)	0	0.19 (0.46)
	3	200	0	0.19 (0.44)	0	0.01 (0.1)	0	0.07 (0.26)	0	0.12 (0.35)	0	0.01 (0.1)	0	0.09 (0.29)	0	0.04 (0.2)	0	0.12 (0.35)
		1000	0	0.27 (0.49)	0	0.0 (0.0)	0	0.12 (0.32)	0	0.11 (0.34)	0	0.05 (0.22)	0	0.14 (0.37)	0	0.11 (0.31)	0	0.17 (0.4)

		2000	10	0.54 (0.65)	0	0.03 (0.17)	8	0.35 (0.52)	7	0.29 (0.53)	4	0.17 (0.38)	4	0.31 (0.58)	6	0.27 (0.47)	10	0.43 (0.53)
		3000	12	0.56 (0.61)	3	0.12 (0.32)	8	0.25 (0.46)	10	0.43 (0.53)	5	0.16 (0.39)	11	0.38 (0.54)	7	0.25 (0.43)	12	0.43 (0.57)

TP and FP stand for true and false positive detections respectively. True positive detections are reported as the total number of accurately identified recombination events across all simulations, whereas the values indicated for false detection are the mean value per replicate along with the standard deviation. The column "All" reports the TP and FP values across all detection methods.

Table G. Results of the RDP4 analyses carried on one hundred simulated datasets with recombination at detection *P*-value of 1.0E-9 and requiring the agreement between at least two methods.

	event	Recombination length	Method															
			All		3Seq		Bootscan		Chimaera		GENECONV		Maxchi		RDP		SiSscan	
			TP	FP	TP	FP	TP	FP	TP	FP	TP	FP	TP	FP	TP	FP	TP	FP
Subtype FE	1	200	0	0.01 (0.1)	0	0.0 (0.0)	0	0.01 (0.1)	0	0.0 (0.0)	0	0.0 (0.0)	0	0.0 (0.0)	0	0.01 (0.1)	0	0.0 (0.0)
		1000	37	0.0 (0.0)	11	0.0 (0.0)	36	0.0 (0.0)	13	0.0 (0.0)	7	0.0 (0.0)	13	0.0 (0.0)	37	0.0 (0.0)	8	0.0 (0.0)
		2000	49	0.01 (0.1)	43	0.01 (0.1)	43	0.0 (0.0)	40	0.0 (0.0)	6	0.0 (0.0)	45	0.0 (0.0)	48	0.0 (0.0)	36	0.01 (0.1)
		3000	49	0.03 (0.17)	46	0.02 (0.14)	36	0.01 (0.1)	39	0.01 (0.1)	11	0.0 (0.0)	48	0.01 (0.1)	43	0.02 (0.14)	38	0.02 (0.14)
	2	200	0	0.0 (0.0)	0	0.0 (0.0)	0	0.0 (0.0)	0	0.0 (0.0)	0	0.0 (0.0)	0	0.0 (0.0)	0	0.0 (0.0)	0	0.0 (0.0)
		1000	63	0.0 (0.0)	4	0.0 (0.0)	63	0.0 (0.0)	5	0.0 (0.0)	26	0.0 (0.0)	3	0.0 (0.0)	62	0.0 (0.0)	26	0.0 (0.0)
		2000	56	0.0 (0.0)	42	0.0 (0.0)	47	0.0 (0.0)	42	0.0 (0.0)	9	0.0 (0.0)	48	0.0 (0.0)	51	0.0 (0.0)	36	0.0 (0.0)
		3000	74	0.06 (0.24)	69	0.05 (0.22)	56	0.01 (0.1)	64	0.02 (0.14)	32	0.0 (0.0)	70	0.04 (0.2)	68	0.01 (0.1)	58	0.04 (0.2)
	3	200	0	0.01 (0.1)	0	0.0 (0.0)	0	0.0 (0.0)	0	0.01 (0.1)	0	0.0 (0.0)	0	0.01 (0.1)	0	0.0 (0.0)	0	0.0 (0.0)
		1000	0	0.0 (0.0)	0	0.0 (0.0)	0	0.0 (0.0)	0	0.0 (0.0)	0	0.0 (0.0)	0	0.0 (0.0)	0	0.0 (0.0)	0	0.0 (0.0)
		2000	0	0.0 (0.0)	0	0.0 (0.0)	0	0.0 (0.0)	0	0.0 (0.0)	0	0.0 (0.0)	0	0.0 (0.0)	0	0.0 (0.0)	0	0.0 (0.0)
		3000	0	0.0 (0.0)	0	0.0 (0.0)	0	0.0 (0.0)	0	0.0 (0.0)	0	0.0 (0.0)	0	0.0 (0.0)	0	0.0 (0.0)	0	0.0 (0.0)
	4	200	5	0.02 (0.14)	0	0.0 (0.0)	5	0.01 (0.1)	0	0.0 (0.0)	5	0.01 (0.1)	0	0.0 (0.0)	4	0.02 (0.14)	0	0.01 (0.1)
		1000	92	0.06 (0.24)	58	0.02 (0.14)	89	0.06 (0.24)	70	0.04 (0.2)	80	0.04 (0.2)	59	0.03 (0.17)	92	0.06 (0.24)	69	0.03 (0.17)
		2000	94	0.02 (0.14)	94	0.02 (0.14)	90	0.01 (0.1)	87	0.02 (0.14)	88	0.01 (0.1)	93	0.02 (0.14)	94	0.02 (0.14)	93	0.02 (0.14)
		3000	98	0.02 (0.14)	98	0.02 (0.14)	98	0.02 (0.14)	95	0.02 (0.14)	95	0.0 (0.0)	98	0.02 (0.14)	98	0.02 (0.14)	98	0.02 (0.14)
Subtype S	1	200	0	0.05 (0.22)	0	0.01 (0.1)	0	0.01 (0.1)	0	0.03 (0.17)	0	0.01 (0.1)	0	0.02 (0.14)	0	0.02 (0.14)	0	0.04 (0.2)
		1000	0	0.22 (0.46)	0	0.04 (0.24)	0	0.16 (0.39)	0	0.1 (0.36)	0	0.01 (0.1)	0	0.06 (0.24)	0	0.17 (0.4)	0	0.1 (0.3)
		2000	0	0.3 (0.54)	0	0.16 (0.42)	0	0.19 (0.44)	0	0.17 (0.4)	0	0.06 (0.24)	0	0.17 (0.4)	0	0.19 (0.44)	0	0.17 (0.43)
		3000	14	0.28 (0.47)	10	0.11 (0.31)	4	0.13 (0.34)	11	0.18 (0.41)	1	0.01 (0.1)	8	0.22 (0.44)	10	0.15 (0.36)	6	0.14 (0.37)
	2	200	0	0.04 (0.24)	0	0.01 (0.1)	0	0.01 (0.1)	0	0.02 (0.14)	0	0.0 (0.0)	0	0.01 (0.1)	0	0.0 (0.0)	0	0.03 (0.22)
		1000	1	0.28 (0.47)	0	0.1 (0.33)	1	0.25 (0.46)	0	0.12 (0.32)	1	0.12 (0.35)	0	0.11 (0.34)	1	0.26 (0.46)	0	0.18 (0.41)
		2000	0	0.51 (0.54)	0	0.29 (0.45)	0	0.41 (0.51)	0	0.33 (0.47)	0	0.1 (0.3)	0	0.31 (0.46)	0	0.45 (0.52)	0	0.28 (0.49)
		3000	30	0.43 (0.51)	27	0.34 (0.49)	16	0.27 (0.47)	25	0.36 (0.5)	9	0.13 (0.36)	28	0.35 (0.5)	25	0.31 (0.48)	22	0.34 (0.49)
	3	200	0	0.02 (0.14)	0	0.01 (0.1)	0	0.0 (0.0)	0	0.0 (0.0)	0	0.0 (0.0)	0	0.01 (0.1)	0	0.0 (0.0)	0	0.02 (0.14)
		1000	0	0.15 (0.38)	0	0.01 (0.1)	0	0.1 (0.3)	0	0.05 (0.22)	0	0.03 (0.17)	0	0.03 (0.17)	0	0.1 (0.3)	0	0.09 (0.32)
		2000	0	0.27 (0.44)	0	0.16 (0.37)	0	0.16 (0.37)	0	0.15 (0.36)	0	0.04 (0.2)	0	0.18 (0.38)	0	0.19 (0.39)	0	0.18 (0.38)
		3000	10	0.25 (0.46)	10	0.17 (0.38)	2	0.1 (0.33)	7	0.14 (0.35)	2	0.03 (0.22)	10	0.18 (0.38)	7	0.14 (0.37)	6	0.17 (0.4)
Subtype W	1	200	0	0.0 (0.0)	0	0.0 (0.0)	0	0.0 (0.0)	0	0.0 (0.0)	0	0.0 (0.0)	0	0.0 (0.0)	0	0.0 (0.0)	0	0.0 (0.0)
		1000	0	0.01 (0.1)	0	0.0 (0.0)	0	0.01 (0.1)	0	0.0 (0.0)	0	0.0 (0.0)	0	0.01 (0.1)	0	0.01 (0.1)	0	0.01 (0.1)
		2000	0	0.0 (0.0)	0	0.0 (0.0)	0	0.0 (0.0)	0	0.0 (0.0)	0	0.0 (0.0)	0	0.0 (0.0)	0	0.0 (0.0)	0	0.0 (0.0)
		3000	0	0.01 (0.1)	0	0.0 (0.0)	0	0.01 (0.1)	0	0.0 (0.0)	0	0.0 (0.0)	0	0.0 (0.0)	0	0.01 (0.1)	0	0.01 (0.1)
	2	200	0	0.0 (0.0)	0	0.0 (0.0)	0	0.0 (0.0)	0	0.0 (0.0)	0	0.0 (0.0)	0	0.0 (0.0)	0	0.0 (0.0)	0	0.0 (0.0)
		1000	0	0.01 (0.1)	0	0.01 (0.1)	0	0.0 (0.0)	0	0.0 (0.0)	0	0.0 (0.0)	0	0.0 (0.0)	0	0.0 (0.0)	0	0.01 (0.1)
		2000	0	0.02 (0.14)	0	0.0 (0.0)	0	0.02 (0.14)	0	0.0 (0.0)	0	0.01 (0.1)	0	0.0 (0.0)	0	0.02 (0.14)	0	0.02 (0.14)
		3000	0	0.01 (0.1)	0	0.0 (0.0)	0	0.0 (0.0)	0	0.0 (0.0)	0	0.0 (0.0)	0	0.01 (0.1)	0	0.0 (0.0)	0	0.01 (0.1)
	3	200	0	0.0 (0.0)	0	0.0 (0.0)	0	0.0 (0.0)	0	0.0 (0.0)	0	0.0 (0.0)	0	0.0 (0.0)	0	0.0 (0.0)	0	0.0 (0.0)
		1000	0	0.05 (0.22)	0	0.0 (0.0)	0	0.05 (0.22)	0	0.0 (0.0)	0	0.04 (0.2)	0	0.0 (0.0)	0	0.02 (0.14)	0	0.05 (0.22)

		2000	4	0.15 (0.36)	0	0.01 (0.1)	4	0.15 (0.36)	1	0.02 (0.14)	2	0.06 (0.24)	1	0.05 (0.22)	3	0.09 (0.29)	4	0.12 (0.32)
		3000	8	0.2 (0.4)	2	0.03 (0.17)	6	0.13 (0.34)	6	0.08 (0.27)	5	0.09 (0.29)	3	0.09 (0.29)	5	0.12 (0.32)	8	0.17 (0.38)

TP and FP stand for true and false positive detections respectively. True positive detections are reported as the total number of accurately identified recombination events across all simulations, whereas the values indicated for false detection are the mean value per replicate along with the standard deviation. The column “All” reports the TP and FP values across all detection methods.

Table H. Results of the RDP4 analyses carried on one hundred simulated datasets with recombination at detection *P*-value of 0.05 and requiring the agreement between at least three methods.

	event	Recombination length	Method															
			All		3Seq		Bootscan		Chimaera		GENECONV		Maxchi		RDP		SiSscan	
			TP	FP	TP	FP	TP	FP	TP	FP	TP	FP	TP	FP	TP	FP	TP	FP
Subtype FE	1	200	18	1.52 (1.26)	0	0.15 (0.38)	18	0.92 (0.99)	11	1.2 (1.19)	12	0.15 (0.41)	10	1.11 (1.13)	18	1.0 (0.98)	8	1.12 (1.05)
		1000	68	1.27 (1.27)	25	0.15 (0.38)	57	0.87 (1.07)	65	0.9 (1.09)	27	0.23 (0.44)	63	0.84 (1.1)	60	0.96 (1.11)	60	0.84 (0.98)
		2000	67	1.61 (1.17)	52	0.31 (0.56)	58	0.89 (0.98)	67	1.2 (1.07)	27	0.17 (0.4)	67	1.14 (0.94)	66	1.09 (0.97)	59	1.14 (0.91)
		3000	69	1.64 (1.42)	55	0.19 (0.44)	58	1.08 (1.14)	68	1.12 (1.1)	25	0.2 (0.51)	69	1.08 (1.06)	63	1.26 (1.24)	66	1.19 (1.2)
	2	200	5	1.59 (1.36)	0	0.24 (0.51)	5	0.9 (0.88)	2	1.14 (1.22)	5	0.19 (0.39)	1	1.08 (1.15)	5	0.96 (1.03)	3	1.08 (1.16)
		1000	87	1.37 (1.11)	44	0.18 (0.41)	85	0.76 (0.81)	87	0.94 (0.9)	59	0.24 (0.49)	86	0.93 (0.93)	87	0.92 (0.86)	80	0.95 (0.89)
		2000	81	1.65 (1.26)	57	0.21 (0.45)	71	1.09 (1.04)	77	1.18 (1.13)	34	0.28 (0.51)	80	1.22 (1.11)	75	1.12 (0.99)	77	1.16 (1.09)
		3000	85	1.55 (1.31)	80	0.22 (0.48)	75	0.93 (1.02)	85	1.15 (1.11)	52	0.15 (0.36)	85	1.1 (1.07)	84	1.09 (1.15)	79	1.06 (0.97)
	3	200	0	1.42 (1.33)	0	0.16 (0.44)	0	0.56 (0.83)	0	1.18 (1.3)	0	0.13 (0.34)	0	1.23 (1.24)	0	0.7 (0.89)	0	1.18 (1.16)
		1000	2	1.18 (1.07)	2	0.15 (0.38)	2	0.66 (0.84)	1	0.9 (1.01)	2	0.16 (0.39)	1	0.88 (0.93)	1	0.81 (0.82)	1	0.86 (0.96)
		2000	14	1.44 (1.34)	14	0.2 (0.42)	11	0.94 (1.17)	10	1.05 (1.21)	5	0.22 (0.52)	12	1.07 (1.12)	13	1.01 (1.16)	4	1.06 (1.16)
		3000	11	1.62 (1.21)	11	0.35 (0.55)	7	0.92 (0.92)	11	1.38 (1.07)	6	0.28 (0.55)	11	1.28 (1.11)	9	1.04 (0.96)	6	1.14 (1.07)
	4	200	39	1.71 (1.2)	0	0.19 (0.42)	37	0.91 (0.84)	18	1.24 (1.02)	38	0.38 (0.56)	16	1.26 (1.09)	39	1.18 (0.99)	19	1.31 (1.09)
		1000	93	0.99 (1.04)	85	0.15 (0.38)	89	0.61 (0.84)	93	0.69 (0.92)	92	0.27 (0.47)	93	0.69 (0.92)	93	0.76 (0.84)	91	0.68 (0.82)
		2000	94	1.25 (1.31)	94	0.17 (0.4)	90	0.52 (0.9)	94	1.01 (1.04)	92	0.22 (0.56)	94	1.0 (1.05)	94	0.74 (0.96)	94	0.89 (1.17)
		3000	98	1.36 (1.22)	98	0.21 (0.43)	98	0.76 (0.84)	98	0.96 (1.0)	97	0.17 (0.38)	98	0.98 (1.04)	98	0.84 (0.98)	98	0.97 (1.09)
Subtype S	1	200	2	2.67 (1.45)	0	0.51 (0.69)	2	1.96 (1.36)	1	2.21 (1.38)	1	0.47 (0.62)	1	2.04 (1.33)	2	2.04 (1.31)	1	1.66 (1.16)
		1000	9	3.4 (1.93)	1	0.84 (1.05)	7	2.55 (1.57)	7	2.79 (1.69)	2	0.7 (0.75)	8	2.75 (1.79)	7	2.73 (1.55)	6	2.19 (1.49)
		2000	12	3.18 (1.71)	1	0.9 (1.07)	6	2.42 (1.5)	12	2.77 (1.67)	0	0.66 (0.75)	12	2.75 (1.61)	8	2.71 (1.54)	9	2.16 (1.29)
		3000	40	3.03 (1.66)	28	0.83 (0.95)	24	2.18 (1.41)	39	2.54 (1.42)	9	0.61 (0.75)	38	2.65 (1.58)	37	2.46 (1.47)	34	2.08 (1.41)
	2	200	1	2.7 (1.61)	0	0.49 (0.83)	1	1.86 (1.35)	0	2.09 (1.4)	1	0.6 (0.79)	0	2.03 (1.36)	1	2.06 (1.33)	1	1.78 (1.37)
		1000	5	3.04 (1.48)	3	0.81 (1.01)	4	2.25 (1.27)	5	2.6 (1.4)	3	0.88 (0.84)	5	2.57 (1.33)	5	2.56 (1.44)	3	2.17 (1.3)
		2000	2	3.68 (1.75)	1	1.22 (0.95)	1	2.65 (1.56)	2	3.12 (1.53)	0	0.97 (0.87)	2	2.95 (1.62)	1	2.92 (1.49)	1	2.63 (1.5)
		3000	36	3.05 (1.82)	34	0.98 (1.01)	35	2.13 (1.54)	36	2.54 (1.55)	19	0.81 (0.98)	36	2.47 (1.54)	35	2.41 (1.64)	32	1.92 (1.38)
	3	200	0	2.52 (1.63)	0	0.42 (0.75)	0	1.76 (1.39)	0	1.99 (1.53)	0	0.48 (0.75)	0	1.91 (1.46)	0	1.89 (1.44)	0	1.73 (1.37)
		1000	5	2.99 (1.71)	0	0.66 (0.84)	5	2.15 (1.36)	5	2.45 (1.53)	1	0.65 (0.73)	4	2.45 (1.56)	5	2.32 (1.5)	3	2.05 (1.34)
		2000	7	3.22 (1.51)	2	0.78 (0.83)	4	2.4 (1.36)	6	2.6 (1.39)	1	0.65 (0.7)	7	2.57 (1.39)	5	2.7 (1.35)	4	2.22 (1.43)
		3000	36	3.08 (1.72)	24	0.89 (1.04)	23	2.09 (1.3)	36	2.58 (1.63)	15	0.55 (0.74)	36	2.56 (1.63)	34	2.4 (1.49)	28	1.95 (1.33)
Subtype W	1	200	1	3.78 (1.97)	0	0.52 (0.85)	1	2.2 (1.5)	0	3.05 (1.74)	1	0.79 (0.83)	0	3.13 (1.83)	1	2.62 (1.53)	1	2.79 (1.78)
		1000	12	3.76 (1.98)	0	0.53 (0.83)	10	2.52 (1.62)	9	2.86 (1.95)	6	0.86 (0.97)	7	3.01 (1.92)	10	2.63 (1.62)	10	2.84 (1.64)
		2000	10	3.64 (1.86)	1	0.56 (0.77)	4	2.34 (1.61)	9	2.89 (1.62)	2	0.69 (0.89)	9	2.93 (1.56)	8	2.56 (1.59)	7	2.68 (1.63)
		3000	7	3.96 (2.07)	0	0.63 (0.77)	2	2.57 (1.62)	7	3.14 (1.82)	1	0.97 (0.94)	7	3.18 (1.9)	5	2.57 (1.51)	4	2.9 (1.76)
	2	200	0	3.76 (1.99)	0	0.56 (0.93)	0	2.41 (1.58)	0	2.97 (1.84)	0	0.77 (0.98)	0	2.95 (1.72)	0	2.61 (1.62)	0	2.66 (1.63)
		1000	4	4.04 (2.09)	0	0.63 (1.02)	3	2.51 (1.81)	3	3.24 (1.87)	0	0.74 (0.98)	2	3.22 (1.94)	4	2.7 (1.58)	3	3.11 (1.84)
		2000	4	3.75 (1.68)	3	0.52 (0.85)	0	2.31 (1.21)	4	3.05 (1.67)	0	0.76 (0.92)	4	3.12 (1.72)	3	2.43 (1.25)	2	2.67 (1.3)
		3000	1	3.52 (1.9)	1	0.53 (0.85)	1	2.07 (1.41)	1	2.83 (1.64)	0	0.57 (0.74)	1	2.82 (1.66)	1	2.45 (1.53)	1	2.67 (1.52)
	3	200	0	3.7 (2.02)	0	0.45 (0.79)	0	2.27 (1.39)	0	2.92 (1.85)	0	0.85 (0.85)	0	2.85 (1.71)	0	2.44 (1.56)	0	2.69 (1.55)
		1000	6	3.82 (2.03)	0	0.47 (0.75)	6	2.3 (1.57)	6	3.1 (1.76)	1	0.78 (0.78)	6	3.12 (1.76)	6	2.66 (1.73)	6	2.89 (1.65)

		2000	15	4.48 (2.02)	6	0.77 (0.93)	14	2.98 (1.72)	15	3.55 (1.68)	9	1.24 (1.27)	15	3.7 (1.79)	14	3.19 (1.86)	14	3.38 (1.64)
		3000	19	4.45 (1.97)	9	1.0 (0.97)	17	3.06 (1.68)	19	3.48 (1.77)	9	1.25 (1.17)	19	3.74 (1.77)	18	3.16 (1.69)	19	3.42 (1.82)

TP and FP stand for true and false positive detections respectively. True positive detections are reported as the total number of accurately identified recombination events across all simulations, whereas the values indicated for false detection are the mean value per replicate along with the standard deviation. The column “All” reports the TP and FP values across all detection methods.

Table I. Results of the RDP4 analyses carried on one hundred simulated datasets with recombination at detection *P*-value of 1.0E-6 and requiring the agreement between at least three methods.

	event	Recombination length	Method															
			All		3Seq		Bootscan		Chimaera		GENECONV		Maxchi		RDP		SiSscan	
			TP	FP	TP	FP	TP	FP	TP	FP	TP	FP	TP	FP	TP	FP	TP	FP
Subtype FE	1	200	1	0.02 (0.14)	0	0.0 (0.0)	1	0.01 (0.1)	0	0.01 (0.1)	1	0.01 (0.1)	0	0.01 (0.1)	1	0.01 (0.1)	0	0.02 (0.14)
		1000	40	0.02 (0.14)	12	0.0 (0.0)	37	0.01 (0.1)	35	0.01 (0.1)	12	0.01 (0.1)	28	0.01 (0.1)	40	0.01 (0.1)	27	0.01 (0.1)
		2000	50	0.01 (0.1)	48	0.0 (0.0)	48	0.01 (0.1)	41	0.01 (0.1)	12	0.0 (0.0)	48	0.0 (0.0)	49	0.01 (0.1)	43	0.01 (0.1)
		3000	58	0.02 (0.14)	48	0.0 (0.0)	54	0.02 (0.14)	46	0.01 (0.1)	12	0.01 (0.1)	55	0.01 (0.1)	57	0.01 (0.1)	50	0.01 (0.1)
	2	200	0	0.03 (0.17)	0	0.0 (0.0)	0	0.02 (0.14)	0	0.01 (0.1)	0	0.01 (0.1)	0	0.01 (0.1)	0	0.02 (0.14)	0	0.02 (0.14)
		1000	64	0.04 (0.2)	12	0.0 (0.0)	64	0.03 (0.17)	40	0.02 (0.14)	35	0.0 (0.0)	31	0.01 (0.1)	64	0.03 (0.17)	53	0.04 (0.2)
		2000	58	0.02 (0.14)	48	0.0 (0.0)	54	0.02 (0.14)	46	0.01 (0.1)	12	0.01 (0.1)	55	0.01 (0.1)	57	0.01 (0.1)	50	0.01 (0.1)
		3000	76	0.06 (0.24)	73	0.05 (0.22)	62	0.01 (0.1)	69	0.03 (0.17)	39	0.0 (0.0)	75	0.04 (0.2)	74	0.01 (0.1)	66	0.05 (0.22)
	3	200	0	0.0 (0.0)	0	0.0 (0.0)	0	0.0 (0.0)	0	0.0 (0.0)	0	0.0 (0.0)	0	0.0 (0.0)	0	0.0 (0.0)	0	0.0 (0.0)
		1000	0	0.01 (0.1)	0	0.01 (0.1)	0	0.0 (0.0)	0	0.0 (0.0)	0	0.0 (0.0)	0	0.01 (0.1)	0	0.01 (0.1)	0	0.0 (0.0)
		2000	0	0.03 (0.22)	0	0.0 (0.0)	0	0.03 (0.22)	0	0.02 (0.14)	0	0.0 (0.0)	0	0.01 (0.1)	0	0.03 (0.22)	0	0.03 (0.22)
		3000	3	0.03 (0.17)	3	0.01 (0.1)	3	0.02 (0.14)	1	0.01 (0.1)	1	0.01 (0.1)	0	0.01 (0.1)	3	0.02 (0.14)	1	0.01 (0.1)
	4	200	9	0.05 (0.22)	0	0.0 (0.0)	9	0.05 (0.22)	1	0.01 (0.1)	9	0.03 (0.17)	1	0.01 (0.1)	9	0.05 (0.22)	0	0.02 (0.14)
		1000	93	0.08 (0.27)	72	0.04 (0.2)	89	0.08 (0.27)	87	0.06 (0.24)	85	0.04 (0.2)	85	0.06 (0.24)	93	0.08 (0.27)	86	0.05 (0.22)
		2000	94	0.02 (0.14)	94	0.02 (0.14)	90	0.01 (0.1)	91	0.02 (0.14)	92	0.01 (0.1)	93	0.02 (0.14)	94	0.02 (0.14)	94	0.02 (0.14)
		3000	98	0.02 (0.14)	98	0.02 (0.14)	98	0.02 (0.14)	95	0.02 (0.14)	96	0.0 (0.0)	98	0.02 (0.14)	98	0.02 (0.14)	98	0.02 (0.14)
Subtype S	1	200	0	0.15 (0.38)	0	0.06 (0.24)	0	0.08 (0.27)	0	0.13 (0.36)	0	0.02 (0.14)	0	0.11 (0.31)	0	0.1 (0.3)	0	0.12 (0.32)
		1000	0	0.37 (0.58)	0	0.1 (0.33)	0	0.26 (0.5)	0	0.26 (0.5)	0	0.1 (0.3)	0	0.25 (0.5)	0	0.3 (0.52)	0	0.28 (0.49)
		2000	0	0.49 (0.69)	0	0.22 (0.52)	0	0.3 (0.54)	0	0.35 (0.61)	0	0.14 (0.35)	0	0.39 (0.6)	0	0.36 (0.56)	0	0.37 (0.59)
		3000	16	0.46 (0.62)	12	0.14 (0.35)	6	0.28 (0.53)	13	0.38 (0.6)	2	0.07 (0.26)	16	0.41 (0.58)	13	0.35 (0.59)	14	0.36 (0.56)
	2	200	0	0.15 (0.38)	0	0.01 (0.1)	0	0.07 (0.26)	0	0.12 (0.35)	0	0.02 (0.14)	0	0.12 (0.35)	0	0.1 (0.3)	0	0.13 (0.34)
		1000	1	0.47 (0.62)	0	0.17 (0.4)	1	0.35 (0.57)	0	0.36 (0.56)	1	0.19 (0.42)	0	0.37 (0.56)	1	0.36 (0.56)	0	0.39 (0.58)
		2000	0	0.76 (0.68)	0	0.47 (0.54)	0	0.57 (0.55)	0	0.63 (0.63)	0	0.19 (0.39)	0	0.64 (0.61)	0	0.64 (0.61)	0	0.59 (0.62)
		3000	32	0.59 (0.58)	28	0.38 (0.51)	24	0.37 (0.52)	30	0.52 (0.56)	12	0.21 (0.43)	32	0.51 (0.57)	30	0.42 (0.53)	27	0.47 (0.57)
	3	200	0	0.03 (0.17)	0	0.0 (0.0)	0	0.02 (0.14)	0	0.02 (0.14)	0	0.0 (0.0)	0	0.02 (0.14)	0	0.02 (0.14)	0	0.02 (0.14)
		1000	0	0.25 (0.48)	0	0.05 (0.22)	0	0.2 (0.4)	0	0.17 (0.43)	0	0.09 (0.29)	0	0.18 (0.43)	0	0.19 (0.39)	0	0.21 (0.45)
		2000	0	0.37 (0.5)	0	0.23 (0.42)	0	0.23 (0.42)	0	0.33 (0.49)	0	0.09 (0.29)	0	0.34 (0.49)	0	0.31 (0.46)	0	0.26 (0.46)
		3000	17	0.38 (0.58)	13	0.21 (0.43)	10	0.21 (0.43)	14	0.31 (0.56)	4	0.1 (0.33)	16	0.33 (0.57)	13	0.25 (0.46)	15	0.32 (0.55)
Subtype W	1	200	0	0.02 (0.14)	0	0.0 (0.0)	0	0.02 (0.14)	0	0.02 (0.14)	0	0.0 (0.0)	0	0.02 (0.14)	0	0.02 (0.14)	0	0.01 (0.1)
		1000	0	0.1 (0.33)	0	0.02 (0.14)	0	0.05 (0.26)	0	0.08 (0.31)	0	0.0 (0.0)	0	0.08 (0.31)	0	0.05 (0.22)	0	0.09 (0.29)
		2000	2	0.05 (0.22)	0	0.0 (0.0)	2	0.01 (0.1)	0	0.05 (0.22)	0	0.0 (0.0)	0	0.04 (0.2)	2	0.0 (0.0)	2	0.05 (0.22)
		3000	0	0.04 (0.2)	0	0.0 (0.0)	0	0.02 (0.14)	0	0.01 (0.1)	0	0.01 (0.1)	0	0.03 (0.17)	0	0.03 (0.17)	0	0.04 (0.2)
	2	200	0	0.05 (0.26)	0	0.0 (0.0)	0	0.03 (0.22)	0	0.02 (0.14)	0	0.01 (0.1)	0	0.03 (0.17)	0	0.03 (0.17)	0	0.04 (0.24)
		1000	0	0.05 (0.22)	0	0.02 (0.14)	0	0.02 (0.14)	0	0.05 (0.22)	0	0.0 (0.0)	0	0.04 (0.2)	0	0.02 (0.14)	0	0.04 (0.2)
		2000	0	0.11 (0.37)	0	0.01 (0.1)	0	0.07 (0.29)	0	0.06 (0.24)	0	0.02 (0.2)	0	0.06 (0.24)	0	0.06 (0.28)	0	0.11 (0.37)
		3000	0	0.07 (0.29)	0	0.0 (0.0)	0	0.02 (0.14)	0	0.06 (0.24)	0	0.0 (0.0)	0	0.05 (0.22)	0	0.02 (0.14)	0	0.07 (0.29)
	3	200	0	0.08 (0.31)	0	0.01 (0.1)	0	0.03 (0.17)	0	0.05 (0.22)	0	0.01 (0.1)	0	0.05 (0.22)	0	0.04 (0.2)	0	0.05 (0.22)
		1000	0	0.09 (0.29)	0	0.0 (0.0)	0	0.07 (0.26)	0	0.03 (0.17)	0	0.05 (0.22)	0	0.05 (0.22)	0	0.06 (0.24)	0	0.08 (0.27)

		2000	7	0.3 (0.48)	0	0.03 (0.17)	7	0.27 (0.44)	5	0.19 (0.42)	4	0.16 (0.37)	4	0.2 (0.42)	6	0.25 (0.46)	7	0.27 (0.44)
		3000	10	0.34 (0.49)	3	0.1 (0.3)	7	0.22 (0.44)	10	0.29 (0.45)	5	0.16 (0.39)	10	0.28 (0.45)	7	0.23 (0.42)	10	0.3 (0.48)

TP and FP stand for true and false positive detections respectively. True positive detections are reported as the total number of accurately identified recombination events across all simulations, whereas the values indicated for false detection are the mean value per replicate along with the standard deviation. The column “All” reports the TP and FP values across all detection methods.

Table J. Results of the RDP4 analyses carried on one hundred simulated datasets with recombination at detection *P*-value of 1.0E-9 and requiring the agreement between at least three methods.

	event	Recombination length	Method															
			All		3Seq		Bootscan		Chimaera		GENECONV		Maxchi		RDP		SiSscan	
			TP	FP	TP	FP	TP	FP	TP	FP	TP	FP	TP	FP	TP	FP	TP	FP
Subtype FE	1	200	0	0.0 (0.0)	0	0.0 (0.0)	0	0.0 (0.0)	0	0.0 (0.0)	0	0.0 (0.0)	0	0.0 (0.0)	0	0.0 (0.0)	0	0.0 (0.0)
		1000	18	0.0 (0.0)	11	0.0 (0.0)	17	0.0 (0.0)	13	0.0 (0.0)	7	0.0 (0.0)	13	0.0 (0.0)	18	0.0 (0.0)	8	0.0 (0.0)
		2000	47	0.0 (0.0)	43	0.0 (0.0)	42	0.0 (0.0)	39	0.0 (0.0)	6	0.0 (0.0)	44	0.0 (0.0)	47	0.0 (0.0)	36	0.0 (0.0)
		3000	46	0.01 (0.1)	45	0.01 (0.1)	36	0.0 (0.0)	37	0.01 (0.1)	11	0.0 (0.0)	46	0.01 (0.1)	43	0.01 (0.1)	37	0.01 (0.1)
	2	200	0	0.0 (0.0)	0	0.0 (0.0)	0	0.0 (0.0)	0	0.0 (0.0)	0	0.0 (0.0)	0	0.0 (0.0)	0	0.0 (0.0)	0	0.0 (0.0)
		1000	37	0.0 (0.0)	4	0.0 (0.0)	37	0.0 (0.0)	5	0.0 (0.0)	25	0.0 (0.0)	3	0.0 (0.0)	37	0.0 (0.0)	26	0.0 (0.0)
		2000	49	0.0 (0.0)	40	0.0 (0.0)	46	0.0 (0.0)	39	0.0 (0.0)	9	0.0 (0.0)	45	0.0 (0.0)	47	0.0 (0.0)	35	0.0 (0.0)
		3000	71	0.04 (0.2)	69	0.04 (0.2)	56	0.0 (0.0)	62	0.02 (0.14)	32	0.0 (0.0)	68	0.04 (0.2)	67	0.0 (0.0)	57	0.03 (0.17)
	3	200	0	0.0 (0.0)	0	0.0 (0.0)	0	0.0 (0.0)	0	0.0 (0.0)	0	0.0 (0.0)	0	0.0 (0.0)	0	0.0 (0.0)	0	0.0 (0.0)
		1000	0	0.0 (0.0)	0	0.0 (0.0)	0	0.0 (0.0)	0	0.0 (0.0)	0	0.0 (0.0)	0	0.0 (0.0)	0	0.0 (0.0)	0	0.0 (0.0)
		2000	0	0.0 (0.0)	0	0.0 (0.0)	0	0.0 (0.0)	0	0.0 (0.0)	0	0.0 (0.0)	0	0.0 (0.0)	0	0.0 (0.0)	0	0.0 (0.0)
		3000	0	0.0 (0.0)	0	0.0 (0.0)	0	0.0 (0.0)	0	0.0 (0.0)	0	0.0 (0.0)	0	0.0 (0.0)	0	0.0 (0.0)	0	0.0 (0.0)
	4	200	4	0.01 (0.1)	0	0.0 (0.0)	4	0.01 (0.1)	0	0.0 (0.0)	4	0.01 (0.1)	0	0.0 (0.0)	4	0.01 (0.1)	0	0.0 (0.0)
		1000	86	0.05 (0.22)	58	0.02 (0.14)	83	0.05 (0.22)	70	0.04 (0.2)	80	0.04 (0.2)	59	0.03 (0.17)	86	0.05 (0.22)	69	0.03 (0.17)
		2000	94	0.02 (0.14)	94	0.02 (0.14)	90	0.01 (0.1)	87	0.02 (0.14)	88	0.01 (0.1)	93	0.02 (0.14)	94	0.02 (0.14)	93	0.02 (0.14)
		3000	98	0.02 (0.14)	98	0.02 (0.14)	98	0.02 (0.14)	95	0.02 (0.14)	95	0.0 (0.0)	98	0.02 (0.14)	98	0.02 (0.14)	98	0.02 (0.14)
Subtype S	1	200	0	0.02 (0.14)	0	0.01 (0.1)	0	0.01 (0.1)	0	0.01 (0.1)	0	0.01 (0.1)	0	0.01 (0.1)	0	0.01 (0.1)	0	0.02 (0.14)
		1000	0	0.13 (0.39)	0	0.04 (0.24)	0	0.09 (0.32)	0	0.08 (0.34)	0	0.01 (0.1)	0	0.04 (0.2)	0	0.11 (0.34)	0	0.09 (0.29)
		2000	0	0.24 (0.47)	0	0.14 (0.37)	0	0.16 (0.39)	0	0.15 (0.38)	0	0.06 (0.24)	0	0.15 (0.38)	0	0.18 (0.41)	0	0.15 (0.38)
		3000	8	0.18 (0.41)	8	0.1 (0.3)	3	0.1 (0.3)	8	0.14 (0.37)	1	0.01 (0.1)	5	0.16 (0.39)	8	0.12 (0.32)	5	0.11 (0.34)
	2	200	0	0.0 (0.0)	0	0.0 (0.0)	0	0.0 (0.0)	0	0.0 (0.0)	0	0.0 (0.0)	0	0.0 (0.0)	0	0.0 (0.0)	0	0.0 (0.0)
		1000	1	0.2 (0.42)	0	0.09 (0.32)	1	0.19 (0.42)	0	0.11 (0.31)	1	0.12 (0.35)	0	0.1 (0.33)	1	0.2 (0.42)	0	0.17 (0.4)
		2000	0	0.39 (0.53)	0	0.26 (0.44)	0	0.36 (0.5)	0	0.29 (0.45)	0	0.1 (0.3)	0	0.29 (0.45)	0	0.36 (0.5)	0	0.27 (0.49)
		3000	27	0.36 (0.5)	26	0.32 (0.49)	16	0.25 (0.46)	22	0.32 (0.49)	9	0.13 (0.36)	26	0.32 (0.49)	25	0.29 (0.48)	22	0.33 (0.49)
	3	200	0	0.0 (0.0)	0	0.0 (0.0)	0	0.0 (0.0)	0	0.0 (0.0)	0	0.0 (0.0)	0	0.0 (0.0)	0	0.0 (0.0)	0	0.0 (0.0)
		1000	0	0.05 (0.22)	0	0.01 (0.1)	0	0.04 (0.2)	0	0.02 (0.14)	0	0.02 (0.14)	0	0.02 (0.14)	0	0.05 (0.22)	0	0.05 (0.22)
		2000	0	0.19 (0.39)	0	0.14 (0.35)	0	0.13 (0.34)	0	0.14 (0.35)	0	0.04 (0.2)	0	0.17 (0.38)	0	0.14 (0.35)	0	0.14 (0.35)
		3000	10	0.18 (0.41)	10	0.13 (0.34)	2	0.1 (0.33)	7	0.11 (0.31)	2	0.03 (0.22)	10	0.15 (0.36)	7	0.13 (0.36)	6	0.14 (0.37)
Subtype W	1	200	0	0.0 (0.0)	0	0.0 (0.0)	0	0.0 (0.0)	0	0.0 (0.0)	0	0.0 (0.0)	0	0.0 (0.0)	0	0.0 (0.0)	0	0.0 (0.0)
		1000	0	0.01 (0.1)	0	0.0 (0.0)	0	0.01 (0.1)	0	0.0 (0.0)	0	0.0 (0.0)	0	0.01 (0.1)	0	0.01 (0.1)	0	0.01 (0.1)
		2000	0	0.0 (0.0)	0	0.0 (0.0)	0	0.0 (0.0)	0	0.0 (0.0)	0	0.0 (0.0)	0	0.0 (0.0)	0	0.0 (0.0)	0	0.0 (0.0)
		3000	0	0.01 (0.1)	0	0.0 (0.0)	0	0.01 (0.1)	0	0.0 (0.0)	0	0.0 (0.0)	0	0.0 (0.0)	0	0.01 (0.1)	0	0.01 (0.1)
	2	200	0	0.0 (0.0)	0	0.0 (0.0)	0	0.0 (0.0)	0	0.0 (0.0)	0	0.0 (0.0)	0	0.0 (0.0)	0	0.0 (0.0)	0	0.0 (0.0)
		1000	0	0.0 (0.0)	0	0.0 (0.0)	0	0.0 (0.0)	0	0.0 (0.0)	0	0.0 (0.0)	0	0.0 (0.0)	0	0.0 (0.0)	0	0.0 (0.0)
		2000	0	0.02 (0.14)	0	0.0 (0.0)	0	0.02 (0.14)	0	0.0 (0.0)	0	0.01 (0.1)	0	0.0 (0.0)	0	0.02 (0.14)	0	0.02 (0.14)
		3000	0	0.0 (0.0)	0	0.0 (0.0)	0	0.0 (0.0)	0	0.0 (0.0)	0	0.0 (0.0)	0	0.0 (0.0)	0	0.0 (0.0)	0	0.0 (0.0)
	3	200	0	0.0 (0.0)	0	0.0 (0.0)	0	0.0 (0.0)	0	0.0 (0.0)	0	0.0 (0.0)	0	0.0 (0.0)	0	0.0 (0.0)	0	0.0 (0.0)
		1000	0	0.05 (0.22)	0	0.0 (0.0)	0	0.05 (0.22)	0	0.0 (0.0)	0	0.04 (0.2)	0	0.0 (0.0)	0	0.02 (0.14)	0	0.05 (0.22)

		2000	3	0.1 (0.3)	0	0.01 (0.1)	3	0.1 (0.3)	1	0.02 (0.14)	2	0.06 (0.24)	1	0.05 (0.22)	3	0.08 (0.27)	3	0.08 (0.27)
		3000	7	0.13 (0.34)	2	0.03 (0.17)	6	0.12 (0.32)	6	0.05 (0.22)	5	0.09 (0.29)	3	0.05 (0.22)	4	0.11 (0.31)	7	0.12 (0.32)

TP and FP stand for true and false positive detections respectively. True positive detections are reported as the total number of accurately identified recombination events across all simulations, whereas the values indicated for false detection are the mean value per replicate along with the standard deviation. The column “All” reports the TP and FP values across all detection methods.
